# Environmental enrichment as an immunostimulant for rainbow trout aquaculture

**DOI:** 10.1038/s41598-026-44702-2

**Published:** 2026-04-09

**Authors:** Parasuraman Aiya Subramani, Maria Anna Gennaraki, Nasim Emami, Michael Gabel, Hendrik Schubert, Maj-Britt Hölzel, Stefan Reiser, Jörn Peter Scharsack

**Affiliations:** 1 Thünen Institute of Fisheries Ecology, Johann Heinrich von Thünen Institute Herwigstraße 31, 27572 Bremerhaven, Germany; 2 Department of Biology/Chemistry, University of Bremen, Bibliothekstraße 1, 28359 Bremen, Germany

**Keywords:** Immune modulation, Reactive oxygen species, Structural enrichment, Head kidney leukocytes, Respiratory burst, Immunology, Physiology, Zoology

## Abstract

Environmental enrichment (EE) offers a non-invasive strategy to enhance fish welfare, yet its immunological benefits in fish remain incompletely explored and rarely applied in aquaculture practice. This study assessed whether EE exposure alone can modulate immune responses in rainbow trout (*Oncorhynchus mykiss*). EE was implemented as simple physical structures added to otherwise barren tanks, either as horizontally placed gravel substrate (H) simulating a riverbed or vertically suspended rubber cords (V) simulating plant like structures. Tanks without enrichment served as barren controls (C). In experiment 1 (Exp1), trout reared with H, or V enrichment were compared to C for behavioural, neuroendocrine, and immune parameters. The H setup increased social interactions and showed signs of inflammatory activation in the head kidney leukocyte (HKL) functional assays. The V setup led to reduced monocyte counts and slightly higher spontaneous respiratory burst activity (SRBA), although the latter was not statistically significant (*p* > 0.05). In the subsequent experiment using a 2 × 2 factorial design combining enrichment and high (× 2) stocking density (Exp2), trout in the V setup maintained significantly higher immune activity (evident through elevated SRBA, cellular peroxidase content in the HKL, and serum lysozyme activity) under × 2 stocking density conditions (*p* < 0.05). In both experiments, the coefficient of variation (CV) for serum cortisol was higher in the V setup, including at × 2 stocking density, but not significantly different from the C setup (*p* > 0.05). Behavioural modulation was indirectly evidenced by a negative correlation between cortisol levels and body size, suggesting hierarchy formation. Overall, EE influenced neuroendocrine, behavioural, and immune parameters in trout, with V emerging as a practical immunostimulatory measure suitable for juveniles (< 1 year; 5–50 g).

**Supplementary Information:**

The online version contains supplementary material available at 10.1038/s41598-026-44702-2.

## Introduction

Aquaculture is now the leading source of fish production globally, surpassing wild capture fisheries^1^. With increasing consumer demand, the industry faces growing scrutiny regarding the welfare of farmed fish. Public perception of aquaculture-raised fish remains largely negative^2^, underscoring the need for demonstrable welfare improvements to meet both ethical and market expectations. Ensuring high welfare standards is not only crucial for ethical farming but also vital for the sustainability and public image of the aquaculture sector^3^.

Among farmed fish species, rainbow trout (*Oncorhynchus mykiss*, Walbaum 1792) is one of the most extensively cultivated aquaculture species and is also a commonly used model organism^1^. The sentience of trout and other fish species, though debated^4^, warrants careful consideration of their welfare throughout the production cycle^5^. Trout possess a well-developed immune system and are amenable to both behavioural and physiological assessments, making them ideal models for studying the effects of rearing conditions on immune and stress responses^6^. Trout head kidney, an essential immune organ that produces macrophages, monocytes, and lymphocytes can be used to investigate nonspecific cellular immune mechanisms^7^.

Fish welfare encompasses more than physical health: it includes environmental quality and the ability to express natural behaviours^8,9^. Researchers argue that welfare should not only aim to reduce negative experiences but also promote positive ones^5^. Yet, conventional barren aquaculture systems often lack such positive stimulation, contributing to chronic stress in farmed fish^10^. Unlike their wild counterparts, farmed fish cannot escape suboptimal conditions and must continuously cope with behavioural and immunological challenges within their barren and unstimulating environments. Stress in fish is typically assessed by measuring cortisol levels, a widely used physiological indicator^8,11^.

Cortisol, the principal stress hormone in fish, mediates the link between the neuroendocrine and immune systems via the hypothalamic-pituitary-interrenal (HPI) axis^12^. Cortisol plays an important role in behavioural hierarchy of fish with subordinate fish expressing more serum cortisol^13^. While short-term cortisol release helps maintain homeostasis, prolonged systemic elevation may lead to immunosuppression^14^, increased disease susceptibility, reduced growth^15^, and impaired learning^16^. Cortisol responses in fish are highly dynamic and sensitive to handling and sampling timing, an important consideration when interpreting stress-related endocrine and immune responses^17^.

Innate immune mechanisms constitute the first line of defence in fish and are sensitive to changes in stress physiology and welfare conditions. Under favourable rearing environments, these mechanisms can be functionally enhanced, contributing to improved immune competence and disease resistance^3,18^.

Environmental enrichment (EE) refers to the deliberate modification of rearing environments by adding physical, sensory, or social stimuli to simulate natural conditions^19^. Originally developed for zoo and laboratory animals^20^, EE is being repeatedly recommended for aquaculture to reduce stress, promote natural behaviours, and enhance welfare^21^. EE has been shown to reduce cortisol levels^22^, increase brain cell proliferation^23,24^, and alter epigenetic markers in trout brains^25^, indicating its influence on neuroendocrine function.

Beyond enhancing welfare of farmed fish, EE must demonstrate measurable benefits, such as immunostimulation, to ensure its practical relevance and long-term adoption in aquaculture. This is particularly important given that diseases remain one of the most significant constraints to aquaculture growth^26^. Currently, the industry addresses disease challenges through prophylactic approaches such as vaccination, immunostimulants, and probiotics. However, these methods come with limitations: vaccines, for instance, may induce stress^27^ and do not alleviate the underlying welfare concerns in farmed fish^19^.

In addition to behavioural and welfare modulations, EE has been reported to enhance survival^28,29^, reduce pathogen burden^30^, and stimulate certain immune responses^31,32^. However, while some studies report limited insignificant effects on nonspecific serum immune mechanisms^33^, others suggest that EE may modulate nonspecific cellular immune mechanisms such as respiratory burst and phagocytosis^31^. In addition, EE has been shown to enhance nonspecific cellular immune mechanisms including chemotaxis and phagocytosis in a mouse model^34^. These findings prompted an investigation into whether EE stimulates the nonspecific immune response of rainbow trout.

However, recent comprehensive reviews emphasize that physical enrichment in aquaculture is not a uniform intervention, but a context-dependent strategy in which outcomes depend strongly on the specific structural features, spatial configuration, and rearing conditions applied. These works highlight that even minor variations in enrichment design can lead to contrasting behavioural, physiological, and welfare outcomes, underscoring the need to focus on enrichment details rather than enrichment presence alone^35,36^. Within this context, our research focuses on structural enrichment as a practical form of environmental complexity which can be consistently implemented and evaluated under intensive rearing systems.

In our previous study, trout were reared for seven weeks in three EE setups: horizontal enrichment using gravel (H) simulating a riverbed, vertical enrichment using rubber cords (V) simulating plant like structures, and a combination of the two (VH). A barren control (C) setup was also maintained for comparison. These EE configurations were conceptually inspired by natural habitat features encountered by salmonids, such as heterogeneous substrates and vertical structures resembling aquatic vegetation, and were implemented to increase structural complexity rather than to induce exercise or alter water flow. In that study, we found EE to alter leukocyte populations in the head kidney (HK), with the V setup showing lower total leukocyte counts but insignificantly higher SRBA^32^.

In the present study, two experiments (Exp1 and Exp2) were designed to unravel if structural enrichment, specifically the V setup, can stimulate trout immune responses across different stocking densities.

## Materials and methods

### Ethical approval

All experimental procedures were reviewed and approved by the Thünen Institute Animal Welfare Committee, Germany, and the relevant authorities (project no. 42–10; TV Nr. 183 500–427-103–1/2020–4-12).

### Accordance statement

All procedures were performed in accordance with the European Directive 2010/63/EU on the protection of animals used for scientific purposes. The study is reported in accordance with the ARRIVE guidelines^37^.

### Trout husbandry

Experimental trout were obtained from broodstock maintained in circular fiberglass tanks at the aquaculture facility of the Thünen Institute of Fisheries Ecology, Bremerhaven, Germany. Fish were randomly distributed into twelve 40 × 40 × 40 cm (total volume = 64 L) experimental glass aquaria (working volume = 60 L each).

In Experiment 1 (Exp1), 240 fingerlings (mean ± SEM: 4.64 ± 0.07 g; ~ 4 months old) were used, while 360 fingerlings (5.87 ± 0.02 g; ~ 6 months old) were used in the high (× 2) stocking density experiment (Exp2). Aquaria were interconnected via a recirculating pump operating at 120 L/h (Exp1) and 240 L/h (Exp2). Tap water was continuously supplied to a shared reservoir (which also served as a sump) at 60 L/h in Exp1 and 120 L/h in Exp2, corresponding to approximately two and four full water exchanges per day (200% and 400%, respectively). To meet the increased postprandial oxygen demand in Exp2, pure oxygen was introduced into the sump for 15 min daily at 1530 h CET via a compressed gas cylinder.

Trout were fed commercial pellets (Aller Aqua, Christiansfeld, Denmark) at 2% body weight per day, split into two feedings at 1100- and 1400-h CET. Stocking densities and feeding protocols were based on our previous study, which ensured optimal rearing conditions and water quality.

Exp1 examined three rearing conditions: H, V, and a C. Each condition was replicated four times with 20 fish per aquarium. Enrichment setups were previously described^32^. The enrichment configurations were implemented to increase physical complexity and spatial heterogeneity relative to barren aquaria and thus represent forms of physical EE.

Briefly, the H setup consisted of a single layer of gravel (Ø16–32 mm) covering the aquarium bottom, as per Reiser et al.^38^, simulating a riverbed. The V setup contained vertically suspended food-grade EPDM rubber cords (Ø3 mm), spanning two-thirds of the aquarium length and half its height, creating three upper sections, simulating plant like structures. The C setup lacked any structural enrichment.

The experiment lasted 60 days. Based on Exp1 results, the V setup was chosen for Exp2, which employed a 2 × 2 factorial design with two stocking densities (standard: 20 fish/aquarium = 120 fish in total; × 2: 40 fish/aquarium = 240 fish in total) and two enrichment levels (barren; vertical). This resulted in four groups: C (20 fish, barren), V (20 fish, vertical), × 2C (40 fish, barren), and × 2 V (40 fish, vertical), each replicated in three aquaria. A conservative two-fold increase in stocking density was applied based on water quality and growth data from Exp1, indicating that increased biomass could be supported without exceeding biofilter capacity.

All aquaria were maintained on a 12:12 h light:dark cycle, with water temperature held at 16.49 ± 0.05 °C using a heating/cooling unit (Aqua Medic, Germany). Water temperature was selected based on pilot husbandry trials conducted under the same facility conditions and within the tolerated thermal range for rainbow trout. Uneaten feed and faeces were siphoned daily. Rubber cords in the V setup were cleaned weekly; however, waste trapped within gravel crevices in the H setup could not be fully removed.

**Fig. 1:**
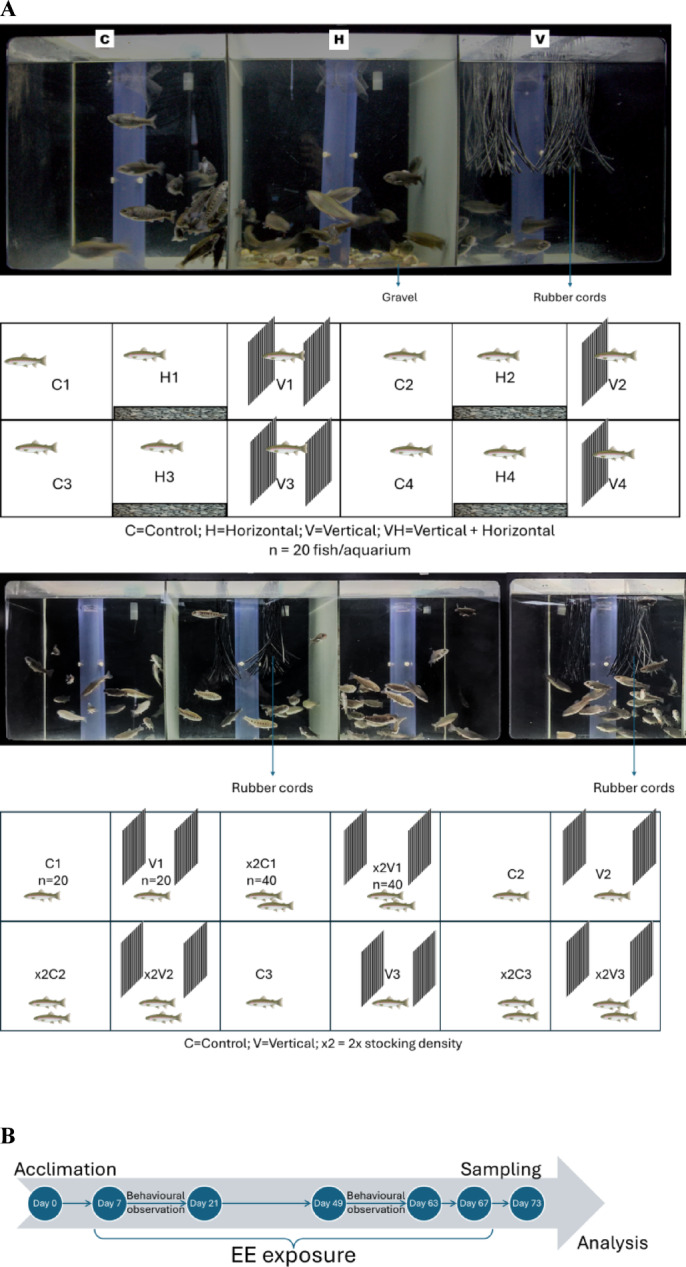
Schematic representation of experimental setup and the timeline. (**A**) illustrates the positioning of environmental enrichment, control setup, and replicates. The inset shows an actual picture of the experimental aquaria. Abbreviations: C = Control, H = Horizontal, V = Vertical, × 2C = high stocking density Control, and × 2 V = high stocking density Vertical. (**B**) Following transfer to experimental aquaria (Day 0), rainbow trout were acclimated for one week before exposure to environmental enrichment. Behavioural observations were conducted during two defined periods (Days 1–14 and Days 42–56). Sampling for behavioural, neuroendocrine, and immune parameters was performed at the end of the 60-day experimental period.

Replicates were assigned continuously, and aquarium arrangement and timeline for both experiments is shown in Fig. 1. Fish were acclimated for one week following transfer to allow recovery from handling; no mortality or abnormal behaviour was observed during this period.

### Water quality

Water quality parameters were measured from the sump that connects all the aquaria three times per week (Monday, Wednesday, and Friday) throughout the experiments. Dissolved oxygen and temperature were measured using a handheld probe (OxyGuard, Farum, Denmark), and pH was recorded using a digital pH meter (Mettler Toledo, Germany). Ammonia, nitrite, and nitrate were quantified using a colorimetric test kit (Merck, Germany). The measured parameters remained well within acceptable ranges for rainbow trout throughout the experiments^39^.

In Exp1, the following water quality parameters were recorded during the enrichment exposure period: dissolved oxygen (9.47 ± 0.02 mg/L), ammonia (0.08 ± 0.01 mg/L), nitrite (0.04 ± 0.01 mg/L), nitrate (2.18 ± 0.15 mg/L), and pH (8.07 ± 0.03). In Exp2, the following water quality parameters were recorded: ammonia (0.12 ± 0.01 mg/L) and nitrite (0.05 ± 0.003 mg/L) levels, along with reduced nitrate (1.89 ± 0.22 mg/L), pH (7.92 ± 0.02), and dissolved oxygen concentrations (8.62 ± 0.09 mg/L).

### Behavioural observations: chasing events

During the first and last two weeks of the experiment, fish in each aquarium were observed for 3 min around 1500 h CET on every weekday, in both experiments. Behavioural observations were conducted by an observer positioned outside the visual field of the aquaria, while video recordings were used only to document representative examples (Supplementary video 1). During observations, the number of chasing events was counted using a click counter. A chasing event was defined as one fish actively pursuing another specimen with rapid movement, often leading to physical contact with or displacement of the chased fish. The number of such interactions was documented for each observation period.

### Sampling

During the 60-day experimental period, mortalities were observed in both experiments, likely due to unknown aetiological factors. In Exp1, the distribution was: C = 5, H = 6, and V = 1. In Exp2, mortalities were noted as follows: C = 4, V = 4, × 2C = 2, and × 2 V = 1 (Supplementary Fig. 1).

At the end of the exposure period, all 228-surviving fish in Exp1 and 232 trout in Exp2 were sampled. All parameters were assessed as end-point measurements following completion of the exposure period. Due to logistical limitations, sampling was performed over six consecutive days, rather than in a single day. This schedule may have introduced temporal variation, which was addressed by including sampling day as a random effect in the statistical model. Additionally, spatial variation arising from the hierarchical experimental structure (multiple replicates across treatments) was accounted for by incorporating both replicate and sampling day as random effects in a generalized linear mixed model (GLMM), following Bolker et al.^40^. Details on model implementation are provided in the statistics section.

On each sampling day, surviving fish from two aquaria were individually netted and were euthanized one by one using an overdose of 2-phenoxyethanol (1 mL/L; v/v; Merck, Darmstadt, Germany). In the × 2C and × 2 V aquaria, all fish were netted using a random sweep method. Every second individual was selected for sampling, whereas the intervening fish were sham handled and immediately transferred to a holding tank. In aquaria where mortality occurred and the number of surviving fish was odd; the final fish was sampled without disrupting the alternating pattern. This approach ensured that no trout was handled twice or subjected to additional stress. All the surviving trout were maintained in accordance with the standard husbandry protocols described above.

For each sampled individual, standard length and body weight were recorded. Tissue samples were collected from the HK, liver, gut, and spleen for downstream analysis. Blood was drawn from the common cardinal vein and allowed to clot at 15 °C in an incubator for 5 h. The serum was then separated by centrifugation at 3000 rpm for 10 min at ambient temperature. This serum processing protocol, as established in our previous study, was found suitable for the analysis of both serum cortisol and immune parameters^41^.

### Growth and Somatic indices

The length and weight of the fish, as well as the weights of the liver, spleen, and gut, were recorded. Additionally, the following indices were calculated based on these measurements:

Weight gain: WG = $\text{final individual weight}-\text{initial average weight per aquarium}$

Specific growth rate: SGR = $100\times \left(\frac{\mathrm{ln}(\text{final individual weight})-\mathrm{ln}(\text{initial average weight})}{60}\right)$

Feed conversion ratio: FCR = $\frac{{feed {\mathrm{int}} ake\dag }}{WG}$

^†Feed intake (2% of body weight, measured in grams) was recorded separately.^

Fulton’s body condition factor: K = $\frac{100 \times \text{ weight of fish}}{{\text{standard length of fish}}^{3}}$

Hepatosomatic index: HSI = $\left(\frac{\text{weight of liver}}{\text{weight of fish}}\right)\times 100$

Gutsomatic index: GutSI = $\left(\frac{\text{weight of gut}}{\text{weight of fish}}\right)\times 100$

Splenosomatic index: SSI = $\left(\frac{\text{weight of spleen}}{\text{weight of fish}}\right)\times 100$

### Serum assays

Serum myeloperoxidase activity was measured using the 3,3′,5,5′-Tetramethylbenzidine (TMB, Sigma-Aldrich, Germany) oxidation method, following our previously described procedures^42^, with modifications based on Quade and Roth^43^. Cortisol levels in trout sera were determined using a commercial competitive ELISA kit (Thermo Scientific, Germany), following the manufacturer’s instructions. Lysozyme activity was assessed via the *Micrococcus lysodeikticus* lysis method (Sigma-Aldrich, Germany) according to our earlier protocol^44^, a modified turbidimetric assay originally described by Ellis^45^.

Heavily haemolysed samples were excluded from all assays. Additionally, trout weighing < 10 g were not bled, and serum samples with volumes below 50 µL were omitted to maintain assay reproducibility. Cortisol analysis was performed on a randomly selected subset of eligible serum samples from each treatment and sampling day. In the cortisol assay, the 96-well microplate format required that 12 wells be reserved for assay standards and blanks, leaving space for a maximum of 84 samples. Furthermore, serum lysozyme activity was measured only in trout from Exp2.

### HKL isolation

All steps were conducted on ice, and all cell culture media, solutions, and buffers were obtained from Carl Roth GmbH, Karlsruhe, Germany, unless otherwise specified. The isolation of HK leukocytes (HKL) from trout was adapted from our established protocol for stickleback^46^. Immediately after dissection, the HK was aseptically transferred to a sterile 40 µm nylon mesh (BD Biosciences, Germany) placed in a Ø 3 cm Petri dish (Greiner Bio, Germany) containing 1 mL of collecting medium (RPMI-1640 supplemented with 20 IU heparin [Sigma-Aldrich, Germany], 100 IU/mL penicillin, and 100 µg/mL streptomycin). The tissue was gently disaggregated using a sterile 2 mL syringe piston (Carl Roth GmbH, Karlsruhe, Germany). Cells remaining in the mesh were rinsed off with an additional 1 mL of collecting medium, and the HKL suspension was transferred to a sterile 15 mL centrifuge tube (BD Biosciences, Germany). HKL were sedimented by centrifugation (600 × g, 4 °C, 5 min). After removing the supernatant, the cells were resuspended and washed once with 2 mL of wash medium (RPMI-1640 supplemented with 10 IU heparin, 100 IU/mL penicillin, and 100 µg/mL streptomycin). Finally, cells were resuspended in 500 µL of plain RPMI-1640 supplemented with 100 IU/mL penicillin and 100 µg/mL streptomycin.

#### Differential counting

Flow cytometry (Beckman Coulter, Krefeld, Germany) was used to quantify HKL counts. A 10 µL aliquot of the HKL suspension was mixed with 60 µL PBS, 20 µL of 10 µg/mL propidium iodide (Sigma-Aldrich, Germany), and 10 µL CytoFLEX Ready-to-Use Daily QC fluorospheres (1013 beads/µL; Beckman Coulter, Krefeld, Germany) in a U-bottom 96-well microtiter plate (Greiner Bio, Germany). Data acquisition continued until 2500 beads were counted or until the 80 µL of sample volume was exhausted. Forward and side scatter dot plots were used for gating, following the approach described in our previous study^32^.

HKL counts were expressed as cells per µL beads. Given that larger fish have larger HKs and based on the observed strong correlation between total cell numbers and body weight data (Spearman ρ = 0.43, *p* < 0.0001, n = 460), the HKL count was normalized by dividing the cell numbers by fish body weight. This normalization yielded the HKL somatic index, calculated as:

$$\text{HKL somatic index }=\frac{\text{Total leukocyte count}}{\text{Weight of fish}}$$

Subpopulations of HKL, namely granulocytes, lymphocytes, and monocytes, were identified through gating. Their counts and differential percentages were obtained directly from the cytometer. Counts were used to calculate somatic indices using the same formula as mentioned above.

#### Cellular peroxidase activity

The peroxidase content of HKL was estimated using the TMB oxidation method, as described in our previous protocol^47^ originally based on the method by Palić et al.^48^. Briefly, 2.5 × 10^6^ HKL per well were added to a U-bottom 96-well plate and lysed with 0.02% Triton X-100 (v/v in phenol red-free Hank’s balanced salt solution containing Mg^2+^ and EGTA; Carl Roth GmbH, Karlsruhe, Germany) to release cellular myeloperoxidase. TMB was added to the lysate to initiate the reaction, which was stopped after 2 min with 1 M HCl. The plate was then centrifuged at 600 × g for 10 min at 4 °C, and the yellow supernatant was transferred to a fresh plate. Finally, the optical density was measured at 450 nm using a spectrophotometer (BMG Labtech GmbH, Ortenberg, Germany).

#### Respiratory burst activity of HKL

A luminometer-based respiratory burst activity assay was conducted following our previously described procedures^32^, modified from Dittmar et al.^49^. Briefly, two sets of 10^6^ HKL/mL from each fish were incubated with lucigenin (250 µg/mL; Sigma-Aldrich, Germany) for 30 min in a flat-bottom white plate (Greiner Bio, Germany) at 20 °C in a CO_2_ incubator. After incubation, one set was treated with 20 µL of zymosan (10 mg/mL; Sigma-Aldrich, Germany), while the other received 20 µL of plain RPMI-1640. Immediately after zymosan addition, the plate was transferred to a luminometer (Berthold, Germany) with a built-in incubator set to 20 °C, and luminescence readings, expressed as relative luminescence units (RLU), were recorded every 5 min for 3 h (36 readouts). The resulting RLU values over time formed an RLU curve, and the area under the RLU curve (RLUarea) was calculated using the trapezoidal rule in ‘R version 4.4.2’^50^ for both stimulated and unstimulated cells.

The stimulation index (SI) was then determined using the formula:

$$\text{SI }=\frac{{\mathrm{RLU}}_{\mathrm{area}} of zymosan treated \left(stimulated\right) cells}{{\mathrm{RLU}}_{\mathrm{area}} of unstimulated cells}$$

#### 2′,7′-Dichlorodihydrofluorescein diacetate (DCFDA) based intracellular reactive oxygen species (ROS) production assay

The DCFDA-based ROS production assay was conducted to measure intracellular ROS content in HKLs. Two sets were prepared: Set 1 contained 59 µL PBS, 20 µL of 2 µg/mL propidium iodide (PI), 10 µL of CytoFLEX Ready-to-Use Daily QC fluorospheres, and 10 µL of an HKL suspension (10^6^ cells/mL) in a 96-well microtiter plate. Set 2 contained the same components, with the addition of 1 µL of 100 µM Phorbol 12-myristate 13-acetate (PMA, Sigma-Aldrich, Germany) prepared in DMSO and a corresponding reduction of PBS to 58 µL. After incubating for 30 min at 20 °C in a CO_2_ incubator, 1 µL of 500 µg/mL DCFDA was added to all wells. The plate was transferred to a Flow Cytometer, where 10,000 live cells (PI-negative) were counted in fast mode. From each aquarium, five fish were randomly selected for analysis. A model DCFDA profile is shown in Supplementary Fig. 2. The procedure was standardized through preliminary experiments for optimal reagent concentrations and incubation times. Geometric mean of DCFDA fluorescence was used for data presentation.

#### Statistics

All statistical analyses were performed using ‘R version 4.4.2’^50^. Data distributions were first visually inspected using histograms and formally assessed using the *fitdistrplus* package^51^.

Chasing behaviour data were analysed using generalized linear models (GLMs) with a Gamma distribution and inverse link function to account for positively skewed continuous data. Group, observation period (first vs last two weeks), and their interaction were included as fixed effects.

For all other endpoints, generalized linear mixed models (GLMMs) were fitted using the *lme4* package^52^. In Exp1, enrichment group (C, H, V) was included as a fixed effect. In Exp2, stocking density (standard, × 2), enrichment (no, yes), and their interaction were included as fixed effects to reflect the 2 × 2 factorial design. Replicate and sampling day were included as random intercepts to account for the hierarchical experimental structure and temporal variation^40^. Response variables were modelled using a Gamma distribution with a log link unless otherwise stated.

Model assumptions were evaluated for all endpoints, including checks for multicollinearity, residual normality, and homoscedasticity. All models included in the analyses met these assumptions. Multicollinearity among fixed effects was assessed using variance inflation factors (VIF) calculated with the *car* package^53^, with VIF values below 5 considered acceptable.

Significance of fixed effects was assessed using likelihood ratio tests based on Chi-squared statistics. For Exp2, Type II or Type III analyses of deviance were applied where appropriate. Post hoc pairwise comparisons were conducted using estimated marginal means with the *emmeans* package^54^. Statistical difference between means was considered significant when *p* < 0.05.

Regression analyses were performed using GLMMs to evaluate associations between physiological parameters (e.g., cortisol) and biometric variables, with replicate and sampling day included as random effects. Confidence intervals for model estimates were obtained using likelihood-based methods. Spearman rank correlations were used where appropriate.

Survival data were analysed using Kaplan–Meier estimators with log-rank (Mantel–Cox) tests implemented in the *survival* package^55^. Survival curves were visualized using the survminer package^56^.

Data are presented as mean ± standard error of the mean (SEM) unless stated otherwise. Graphical representations were generated using *ggplot2*^57^.

## Results

### Mortality

Mortality occurred in all three experimental setups (C = 5, H = 6, V = 1) in Exp1. However, we did not find significant difference in mortality between setups (*p* = 0.16). In Exp2, mortality rates differed significantly between setups (C = 4, V = 4, × 2C = 2, × 2 V = 1; *p* = 0.042), but pairwise comparisons revealed no significant differences (lowest *p* = 0.14 for V: × 2 V). Survival curves of both the experiments were given in Supplementary Fig. 1.

### Behavioural observations: chasing events

Chasing events were enumerated to assess the impact of EE and control setups on aggressive trout behaviour over time. In both C and V setups, the mean number of chasing events significantly decreased during the exposure period. The control setup showed the highest chasing events in the first two weeks and the lowest mean in the last two weeks (estimate: 20.88; *p* < 0.0001). Similarly, in the V setup, the decrease in chasing events was estimated at 10.20 events (*p* = 0.0047). In contrast, the H setup showed a non-significant decrease (estimate: 6.75; *p* = 0.13).

In Exp2, the V setup had no significant effect on the number of chasing events at either standard (estimate: 0.88; *p* = 0.98) or × 2 stocking (estimate: 0.18; *p* = 0.99) density. The standard-density V setup showed the lowest mean number of chasing events during both the initial (20.09 ± 2.1 events) and final weeks (8.48 ± 1.07 events). For all setups, mean chasing events were significantly different between first and the last two weeks (*p* < 0.0001). These temporal trends are illustrated in Supplementary Fig. 3, which shows distinct chasing behaviour patterns across treatments.

### Growth and somatic indices

Among the assessed morphometric indices, only the splenosomatic index (SSI), a proxy for immunologically relevant organ allocation, was significantly reduced in the V setup compared to the C (estimate: C–V = 0.023, *p* = 0.004). No significant difference was detected between the H and V enrichments (Supplementary Fig. 4).

In Exp2, the effects of stocking density and enrichment on the SSI were analysed using estimated marginal means and analysis of deviance. Under enriched conditions, the SSI did not differ between standard and × 2 stocking densities (V vs. × 2 V: E = -0.014, *p* = 0.46). Under barren conditions on the other hand, the SSI was slightly higher in the × 2C setup compared to the C setup (E = -0.033, *p* = 0.086). In contrast to Exp1, within standard stocking density, the SSI was marginally higher in the enriched setup (V) than in the non-enriched C setup (E = 0.013, *p* = 0.09), while no difference was detected at × 2 stocking density (× 2 V vs. × 2C: E = -0.006, *p* = 0.52). Analysis of deviance showed no significant main effects of enrichment or stocking density, although their interaction approached significance (Type II: χ^2^ = 2.91, *p* = 0.088).

Furthermore, growth parameters including initial weight, final weight, weight gain (WG), specific growth rate (SGR), feed conversion ratio (FCR), Fulton’s body condition factor (K), hepatosomatic index (HSI), and gutsomatic index (GutSI)-did not differ significantly across the experimental setups in both Exp1 (Table 1) and the Exp2 (Table 2).

**Table 1:** Growth parameters (mean ± SEM) for the control (C), horizontal (H), and vertical (V) setups (n = 75 for C; n = 74 for H; n = 79 for V).

Parameter	C	H	V
Initial weight (g)	4.42 ± 0.11	4.79 ± 0.12	4.72 ± 0.1
Final weight (g)	18.56 ± 1.01	20.06 ± 0.79	20.84 ± 0.77
Weight gain, WG (g)	14.12 ± 1.03	15.3 ± 0.8	16.13 ± 0.78
Body length, (cm)	11.22 ± 0.24	11.76 ± 0.18	11.91 ± 0.18
Specific growth rate, SGR	2.20 ± 0.10	2.28 ± 0.07	2.37 ± 0.07
Feed conversion ratio, FCR	0.89 ± 0.03	0.86 ± 0.02	0.82 ± 0.01^†^
Fulton’s body condition factor, K (g cm^-3^)	1.25 ± 0.023	1.20 ± 0.019	1.20 ± 0.017
Hepatosomatic index, HSI	1.25 ± 0.04	1.19 ± 0.03	1.21 ± 0.03
Gutsomatic index, GutSI	8.51 ± 0.15	8.38 ± 0.13	8.71 ± 0.16

No significant differences between the means of these parameters (*p* > 0.05). A statistical trend compared to C setup is indicated by † (*p* < 0.1).

**Table 2:** Growth parameters (mean ± SEM) for the control (C), vertical (V), and double stocking density setups (× 2C and × 2 V). Sample sizes: C (n = 56), V (n = 56), × 2C (n = 60), × 2 V (n = 60).

Parameter	C	V	× 2C	× 2 V
Initial weight (g)	5.79 ± 0.06	5.85 ± 0.06	5.9 ± 0.04	5.88 ± 0.04
Final weight (g)	23.69 ± 1.00	23.07 ± 0.72	23.26 ± 0.93	24.76 ± 0.75
Weight gain, WG (g)	17.97 ± 1.97	17.13 ± 0.88	17.35 ± 1.92	18.88 ± 1.21
Body length, (cm)	12.55 ± 0.23	12.46 ± 0.16	12.33 ± 0.22	12.78 ± 0.16
Specific growth rate, SGR	2.34 ± 0.13	2.28 ± 0.08	2.27 ± 0.14	2.39 ± 0.1
Feed conversion ratio, FCR	0.89 ± 0.02	0.93 ± 0.02	0.95 ± 0.05	0.86 ± 0.01
Fulton’s body condition factor, K (g cm^-3^)	1.17 ± 0.021	1.18 ± 0.016	1.23 ± 0.024	1.18 ± 0.017
Hepatosomatic index, HSI	1.46 ± 0.04	1.4 ± 0.04	1.5 ± 0.04	1.46 ± 0.03
Gutsomatic index, GutSI	8.49 ± 0.14	8.72 ± 0.13	8.56 ± 0.19	8.85 ± 0.17

No significant differences were observed between setup means ((*p* > 0.05).

### Serum assays

Serum MPO levels (Supplementary Fig. 5A) were higher in trout exposed to the H setup compared to the V setup after 60 days of exposure; however, this difference is not statistically significant (H–V: E = 0.26, *p* = 0.051). No significant differences were observed between trout in the C and H setups (*p* = 0.21) or between the C and V setups (*p* = 0.84). In Exp2, serum MPO levels did not differ significantly between enrichment or stocking densities.

Lysozyme activity (Supplementary Fig. 5B) was significantly higher in × 2 setups compared to the standard-density setups (χ^2^ = 10.41, *p* = 0.0013), independent of enrichment. Specifically, trout in the × 2 V setup exhibited significantly higher lysozyme activity than those in C (C – × 2 V: E = –42.63, *p* = 0.023), while the × 2C setup also showed elevated activity (C – × 2C: E = –39.19, *p* = 0.038). Under non-enriched conditions, lysozyme activity was significantly higher in the × 2C setup compared to C (E = –39.2, *p* = 0.0075), with a similar trend observed between V and × 2 V under enriched conditions (E = –26.8, *p* = 0.087). However, enrichment itself did not influence lysozyme activity within either stocking density. No significant differences were detected between C and V or between V and × 2 V (*p* > 0.05).

**Fig. 2:**
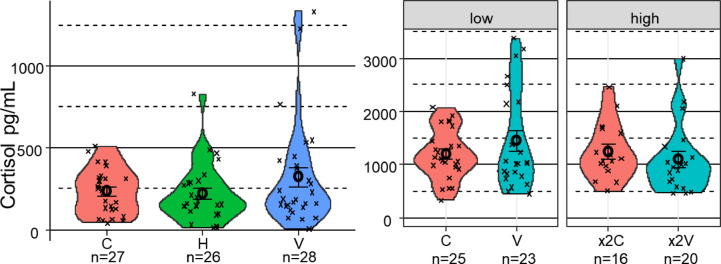
Effect of 60 days of exposure to environmental enrichment setups on serum biomarkers in trout. Serum cortisol content in trout from C, H, and V setups in Exp1 and in trout from the high stocking density Exp2, with control (C), vertical (V), double stocking control (× 2C), and double stocking vertical (× 2 V) setups. No significant differences were observed. Violin plots show the kernel density distribution of raw data, with each point representing an individual fish datum. Mean ± SEM is shown as black circles and error bars. Note: The y-axis scale differs between panels.

The coefficient of variation (CV), a unitless measure, provides a meaningful way to compare relative variability between setups with similar means. Although greater variability was observed within the H and V setups (Fig. 2), cortisol levels did not differ significantly among the C, H, and V setups. The CV, calculated as the ratio between standard deviation and mean, was 1 in the V setup. The lowest p-value (0.14) was noted for the comparison between H and V, with an estimate of –112.5. Under × 2 stocking density, enrichment was associated with a reduction in cortisol levels (E = 361.0, SE = 217, *p* = 0.096), suggesting a modest effect. The CV in cortisol was 0.6 in both the V and × 2 V setups. According to ANOVA, neither enrichment nor stocking density had a significant effect on cortisol levels. The interaction between these two factors was also not statistically significant (χ^2^ = 2.24, *p* = 0.13).

### HKL isolation and differential count

**Fig. 3:**
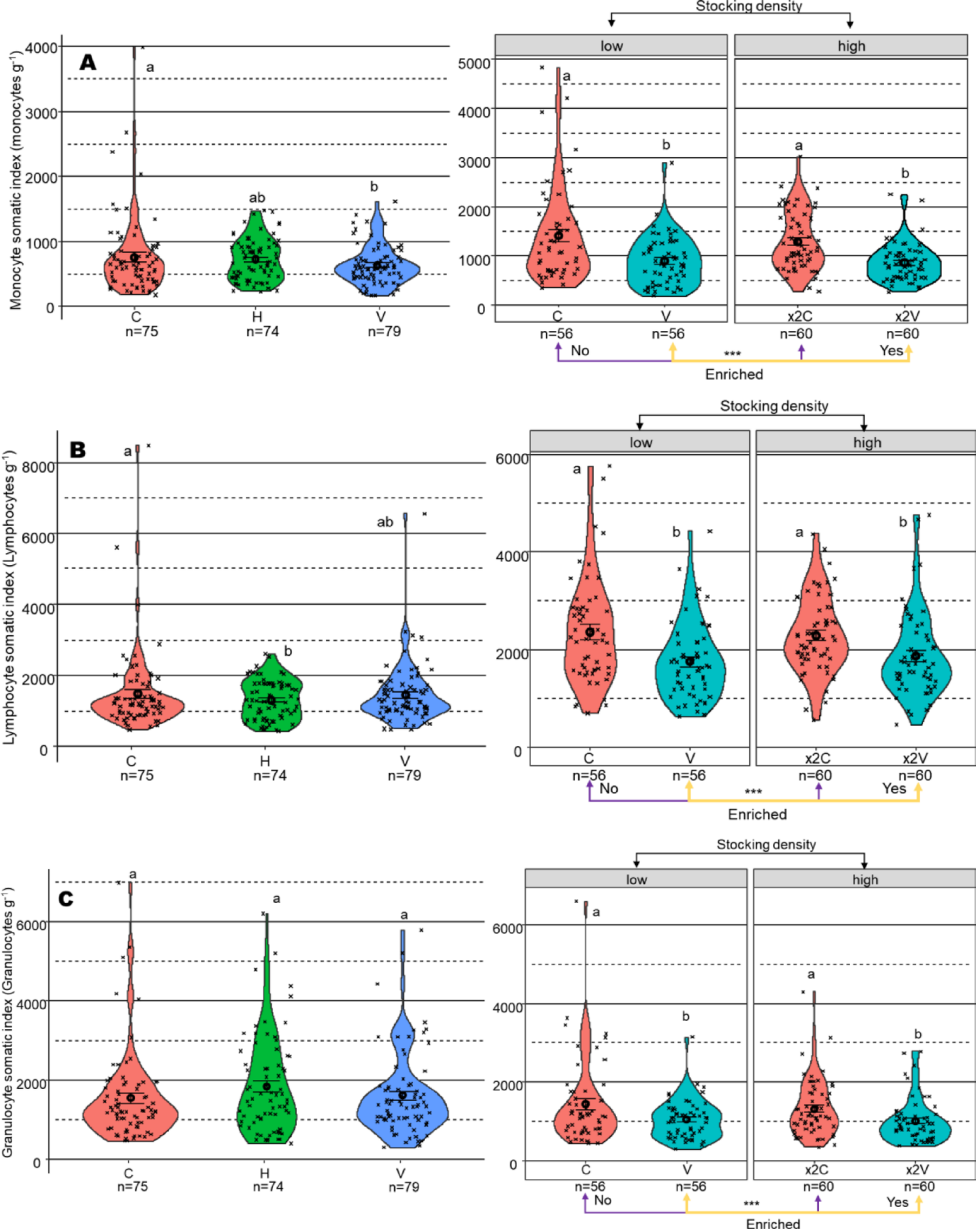
Reduction in head kidney leukocyte (HKL) subpopulations and somatic indices in trout following environmental enrichment. Body weight–normalized HKL counts and leukocyte somatic indices (cells g⁻^1^) were measured in trout exposed for 60 days to control (C), horizontal (H), and vertical (V) enrichment setups in Exp1. In a subsequent × 2 stocking density Exp2, trout were exposed to either C and V setups at standard density or at double stocking density (× 2C, × 2 V). Following exposure, head kidneys were dissected and leukocytes counted using Flow Cytometry; differential counts were obtained by gating. (**A**) Monocyte somatic index, (**B**) lymphocyte somatic index, and (**C**) granulocyte somatic index is shown. Violin plots show kernel density distributions of raw data, with each point representing an individual fish. Mean ± SEM is indicated by black circles and error bars, respectively. Significant differences between factor levels are marked with asterisks (*** for *p* < 0.001), and different letters denote significant differences between setups (*p* < 0.05). Note: The y-axis scale differs between panels.

In Exp1, monocyte counts normalized to fish body weight were significantly reduced in the V setup (Fig. 3A). Compared to the C setup, V setup trout showed a reduction of 489 cells mL⁻^1^ g⁻^1^ (*p* = 0.0005). The difference between H and V was smaller, with V displaying 123 cells mL⁻^1^ g⁻^1^ fewer than H (*p* = 0.0512). In Exp2, a similar enrichment-dependent trend was evident (Fig. 3A). Within both stocking levels, enriched setups (V and × 2 V) had lower monocyte counts than their barren counterparts (C and × 2C). At standard stocking (C vs V), enrichment reduced monocyte levels by 495 cells mL⁻^1^ g⁻^1^ (*p* < 0.0001), and at × 2 stocking (× 2C and × 2 V), by 415 cells mL⁻^1^ g⁻^1^ (*p* = 0.0001). In contrast, stocking level alone did not have a notable effect within either type of enrichment: the difference between standard and × 2 stocking in enriched setups (V and × 2 V) was 24 cells mL⁻^1^ g⁻^1^ (*p* = 0.77), and in barren setups (C and × 2C), 104 cells mL⁻^1^ g⁻^1^ (*p* = 0.41).

Lymphocyte counts normalized to fish body weight followed a similar pattern to that of monocytes, with the lowest values recorded in the H setup (Fig. 3B). Relative to C, lymphocyte abundance dropped by 707 cells mL⁻^1^ g⁻^1^ in H (*p* = 0.039). There was no meaningful difference between C and V (*p* = 0.13) or H and V (*p* = 0.61) setup trout. In Exp2, enrichment again played a more prominent role than stocking level (Fig. 3B). At standard stocking, lymphocyte counts were reduced by 573 cells mL⁻^1^ g⁻^1^ in enriched aquaria compared to barren ones (*p* = 0.0005). A similar reduction of 425 cells mL⁻^1^ g⁻^1^ was observed at × 2 stocking, × 2C and × 2 V trout (*p* = 0.0073). Stocking level, on its own, had no significant effect on lymphocyte counts within either enrichment or barren condition (*p* = 0.6 and *p* = 0.9).

Granulocyte counts normalized to fish body weight were not affected by the experimental setups in Exp1 (Fig. 3C). Upon stepwise deletion, the term *group* did not contribute significantly to the model (*p* = 0.13) which means that the observed differences were not due to enrichment. Further, pairwise contrasts did not indicate any differences between C, H, and V (lowest *p* = 0.13 between C and V). In contrast, enrichment had a clear effect in Exp2. Enriched aquaria exhibited markedly lower granulocyte levels than barren ones, irrespective of stocking density. At standard stocking, enrichment reduced counts by 375 cells mL⁻^1^ g⁻^1^ (*p* = 0.0024), and at × 2 stocking, by 298 cells mL⁻^1^ g⁻^1^ (*p* = 0.0064) compared to their respective barren controls. Stocking level itself had no measurable impact within either enrichment (yes or no) condition (*p* = 0.69 and *p* = 0.37), and the interaction between the two factors was not significant.

A strong positive correlation was observed between HKL granulocyte percentage and cellular responses, including SRBA (ρ = 0.31; *p* < 0.0001, n = 228), zymosan-stimulated respiratory burst (ρ = 0.37; *p* < 0.0001, n = 228), and cellular peroxidase content (ρ = 0.41; *p* < 0.0001, n = 226). In contrast, no clear correlation was found between granulocyte proportion and ROS production in the DCFDA assay. The percentage counts (proportion of each HKL population relative to the total live cells) showed trends comparable to those observed in the absolute counts (data not shown).

### Leukocyte ratios

The granulocyte to lymphocyte ratio (GLR) differed between setups in Exp1 (Supplementary Fig. 6A). The H setup showed a significantly higher ratio compared to C (− 0.36, *p* = 0.0006), while the difference between V and C was negligible (*p* = 0.92). H also exceeded V by 0.32 units (*p* = 0.0031), pointing to a distinct shift in granulocyte–lymphocyte balance under the gravel enrichment. In Exp2, no strong effects were observed. The interaction between enrichment and stocking density was not significant (*p* = 0.29). A marginal trend was noted where standard stocking tended to increase GLR within enriched aquaria (V vs × 2 V) by 0.066 units (*p* = 0.057). No other contrasts were significant (*p* > 0.30).

In Exp1, monocyte to lymphocyte ratio (MLR) varied significantly between the EE setups (Supplementary Fig. 6B). Post hoc comparisons showed that MLR was higher in the H setup compared to the V setup trout (E: 0.1089, *p* = 0.0026). No significant differences were observed between the C and H (*p* = 0.22) or C and V setup trout (*p* = 0.19). In Exp2, analysis of deviance revealed a significant main effect of enrichment on MLR (χ^2^ = 12.62, *p* = 0.00038), while stocking density showed no effect (χ^2^ = 0.064, *p* = 0.80), and the interaction between enrichment and stocking density was non-significant (*p* = 0.99). Pairwise contrasts confirmed that enriched setups had significantly lower MLR compared to non-enriched ones at both standard (E: –0.0773, *p* = 0.022) and × 2 stocking densities (E: –0.0804, *p* = 0.012). Within either enriched or non-enriched conditions, MLR did not differ between standard and × 2 stocking densities (*p* = 0.73 and *p* = 0.83), reinforcing that the enrichment setup, rather than stocking level, modulates monocyte–lymphocyte balance.

### Cellular peroxidase activity

Supplementary Fig. 7A shows, that there were no differences between the setups in Exp1. The interaction between enrichment and stocking level significantly influenced cellular peroxidase activity (Supplementary Fig. 7B; χ^2^ = 6.61, *p* = 0.01). Notably, each factor individually had a significant effect: enrichment (χ^2^ = 7.37, *p* = 0.007) and stocking density (χ^2^ = 21.71, *p* < 0.0001). At standard stocking density, cellular peroxidase was moderately higher in enriched aquaria compared to barren ones (E: 0.15, *p* = 0.02), and this effect became more pronounced at × 2 density (E: 0.44, *p* = 0.0002). Within enriched conditions, differences between stocking levels (× 2 V vs V) were significant (E: − 0.34, *p* = 0.001), whereas no effect was observed between × 2C and C setups, both barren aquaria without enrichment (E: − 0.042, *p* = 0.86). Interestingly, granulocyte percentages were not correlated with cellular peroxidase activity in Exp2 on stocking density.

### Respiratory burst activity of HKL

**Fig. 4:**
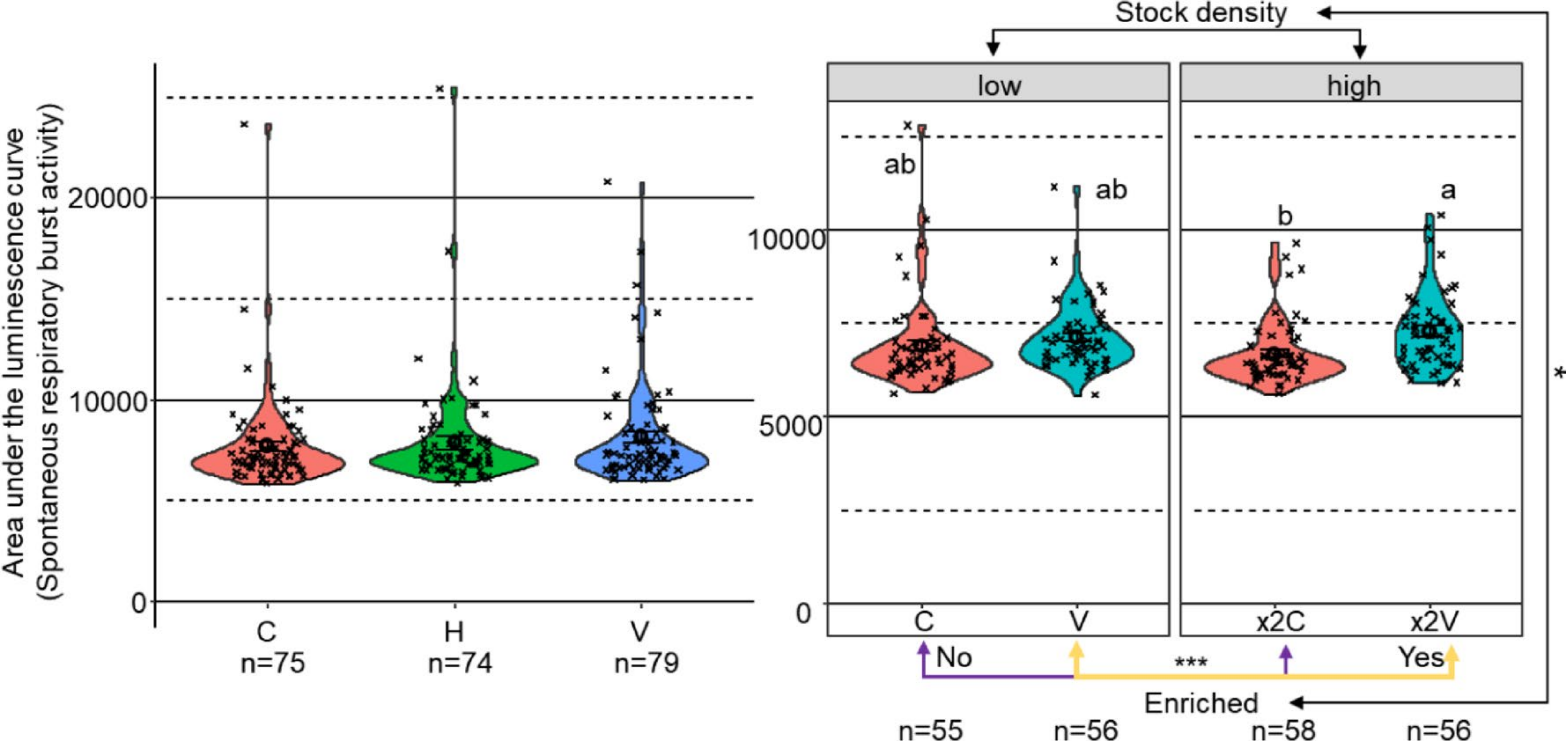
Spontaneous respiratory burst activity (SRBA) in head kidney leukocytes (HKL) of trout from different environmental enrichment setups. Respiratory burst activity was measured in freshly isolated HKL (10⁶ cells/mL) seeded in duplicate in white 96-well luminometer plates. Cells were incubated with lucigenin for 30 min before luminescence measurement. After incubation, luminescence was recorded. Relative light units (RLU) were measured every 5 min for 180 min to generate RLU curves. The area under the curve (RLUarea) was calculated using trapezoidal integration. In Exp1, there were no significant differences between control (C), horizontal (H), and vertical (V) enrichment setups. In Exp2, × 2 V setup trout had significantly higher SRBA compared to the × 2C trout. Violin plots show kernel density of raw values with individual data points. Mean ± SEM is indicated by black circles and error bars. Asterisks denote statistical significance (*** for *p* < 0.001) between the factors (enrichment and stocking density), and different letters indicate significant differences between setup (*p* < 0.05). Note: The y-axis scale differs between panels.

In Exp1, no statistical differences were detected between setups in SRBA (Fig. 4A). Incorporating granulocyte percentages into the model did not improve detection of setup differences, with the lowest p-value observed being 0.19 (C vs V setup). In contrast, in Exp2, trout from the × 2 V setup exhibited significantly higher SRBA compared to × 2C trout (E: 645, *p* = 0.0001; Fig. 4A). Vertical enrichment was strongly associated with this elevated SRBA (χ^2^ = 14.50, *p* = 0.0001). Although the interaction between enrichment and stocking density was marginally significant (χ^2^ = 3.91, *p* = 0.048), stocking density alone did not influence SRBA (*p* = 0.72). Further, addition of granulocyte percentages to the model did not change the results.

There were no differences in the zymosan-stimulated RBA in either experiment (data not shown). In Exp1, the lowest p-value (0.68) was observed between the C and V setups. In Exp2, the comparison between × 2C and × 2 V yielded a p-value of 0.45. When granulocyte percentages were included as a fixed effect, a significant interaction between zymosan RBA and granulocyte percentage was observed (χ^2^ = 29.06, *p* = 0.0001). The corresponding pairwise comparison revealed the lowest p-value (0.26) for the zymosan-stimulated RBA between the C and V setups. In Exp2, the inclusion of granulocyte percentages in the model resulted in an increase in AIC values, making the models unusable.

### DCFDA based ROS production assay

In Exp1 (Supplementary Fig. 8A), trout from the H setup showed a significant increase in intracellular ROS content compared to trout from the C setup (E: 3161, *p* = 0.025). No significant differences were observed between the C and V setups (*p* = 0.36) or between the H and V setups (*p* = 0.54). In Exp2, however, trout from the V setup exhibited higher intracellular ROS content (Supplementary Fig. 8A) than those from the C setup (E: 2902, *p* = 0.0136), although no differences were detected between the × 2 stocking density setups, × 2C and × 2 V (*p* = 0.37). Analysis of deviance (Type II table) indicated that enrichment had a significant effect on intracellular ROS levels (χ^2^ = 5.67, *p* = 0.0173), whereas stocking density did not (*p* = 0.45).

Under PMA stimulation, trout from the H and V setups produced significantly higher levels of intracellular ROS compared to the C setup (Supplementary Fig. 8B). Specifically, H setup trout exhibited an increase of 6571 fluorescence units (*p* = 0.0006), and V setup trout exhibited an increase of 9950 fluorescence units (*p* = 0.0007) relative to C setup trout. However, no difference was observed between the H and V setup trout (*p* = 0.17). In Exp2, PMA stimulation did not induce significant differences in intracellular ROS production between setups, with the lowest p-value observed being 0.39 between the C and V setups.

## Discussion

Aggression in salmonids is well documented, with dominance hierarchies forming through social interactions that influence neuroendocrine pathways^58^. Behavioural observations in the present study revealed that enriched setups modulated trout interactions. In Exp1, the H setup maintained consistent chasing behaviour, while both the C and V setups showed a decline in aggression over time (Supplementary Fig. 4). The emergence of dominant individuals in the enriched environments, who controlled spatial territories and access to resources, indicated the formation of social hierarchies.

In a study on brown trout (*Salmo trutta*), individuals without defined territories segregated into pelagic or demersal forms within a few days of rearing^59^. Consistent with this, the present study observed dominant individuals actively herding subordinates into specific zones, typically one or two quadrants within the aquarium (Supplementary Fig. 9). This observation aligns with previous findings in brown trout and other salmonids, where structural enrichment supported the establishment of territory^60–63^.

Interestingly, in Exp2, overall aggression levels were lower, with the V setup exhibiting the fewest mean chasing events by the end of the trial (*p* > 0.05). Notably, in both experiments, the number of chasing events did not differ significantly between the setups. Owing to the large number of fish in the × 2 stock density aquaria, quadrant-based behavioural analysis was not feasible in Exp2, as frequent overlap of individuals and rapid movement between spatial zones prevented reliable assignment to defined quadrants. More broadly, aquarium size, shape, and internal structure can influence behavioural outcomes by constraining movement and social interactions^35^. Accordingly, while the standardized use of identical aquaria ensured valid relative comparisons among treatments, the behavioural effects of the enrichment structures may be context dependent and could differ in tanks or aquaria with different dimensions, shapes, or spatial configurations.

These behavioural patterns suggest that environmental enrichment influences the formation of social structure and may help reduce aggression. The V setup, in particular, appeared to support spatial stability and encourage natural trout behaviour. While domestication and long-term aquaculture conditions have been suggested to attenuate certain behavioural responses in farmed trout, behavioural plasticity in response to environmental modifications remains evident. The behavioural differences observed here therefore likely reflect context-dependent responses to structural complexity rather than fully instinctive behaviours and remain relevant within aquaculture settings.

Cortisol levels did not significantly differ between setups, but their coefficient of variation (CV) was higher in enriched setups, suggesting variable stress perception and hierarchy effects. A negative correlation between cortisol and body size supports the presence of social stratification, as smaller (subordinate) fish displayed higher cortisol levels, a trend observed in previous studies^64,65^. Notably, cortisol showed weak but significant positive correlations with HKL counts and SRBA, contrasting with the suppressive effects commonly attributed to cortisol on innate immunity^66^. Serum cortisol levels in fish are known to exhibit pronounced diel variation and time-dependent dynamics, characterized by rapid peak responses and subsequent declines driven by homeostatic regulation^67–69^. While such dynamics can complicate single-time-point measurements, in the present study, serum cortisol levels showed consistent correlations with fish morphometrics, supporting the physiological relevance and robustness of the measurements obtained.

While cortisol alone cannot explain the immune modulation observed, these correlations highlight the dynamic interplay between stress physiology and immune function. Enriched environments, especially the V setup, may buffer these effects through structural modulation of behaviour and social dynamics. Additional mechanisms such as epigenetic modifications in the trout brain, as observed in our earlier studies^25^, may also have contributed to the behavioural and neuroendocrine responses.

A key outcome of both experiments was the significant reduction in monocyte and lymphocyte counts in enriched setups, particularly in the V setup. This suggests that enrichment may lead to more efficient immune cell function rather than merely increasing cell numbers. These changes could reflect redistribution of immune cells to peripheral tissues or reduced chronic stress-induced immune depletion^70^. Despite reduced immune cell numbers, functional parameters (SRBA, peroxidase content, and serum lysozyme activity) were significantly enhanced only in the × 2 V setup trout of the Exp2. In both the experiments, V setup trout had a higher mean SRBA but insignificantly. This shift from quantitative to qualitative immune enhancement suggests haematopoietic reprogramming driven by environmental stimuli, potentially involving neuroendocrine and behavioural cues^71,72^.

In a pilot experiment, several fish exhibited deterioration of the caudal fin and abnormal swimming behaviour, symptoms consistent with cold-water disease. These symptoms were not observed when water temperature was increased to 16 °C, which may reflect the temperature-dependent proliferation of *Flavobacterium psychrophilum*, the pathogen commonly associated with cold-water disease in salmonids^73^. In Exp2, daily oxygen supplementation was applied to the shared sump to maintain normoxic conditions and adequate water quality, as commonly practised in aquaculture. Oxygen levels were kept below supersaturation throughout the experiment. Under these conditions, technical oxygenation is unlikely to directly influence immune parameters and therefore does not compromise the interpretation of enrichment-related immune responses under × 2-density conditions.

The H setup exhibited elevated serum myeloperoxidase (MPO), granulocyte-to-lymphocyte (GLR), and monocyte-to-lymphocyte (MLR) ratios, indicating a heightened inflammatory state. Although this may reflect immune readiness, it also carries the risk of oxidative stress and tissue damage^74,75^. In contrast, the V setup achieved high SRBA and peroxidase activity with lower MPO and GLR, pointing to a more balanced and efficient immune state. This was particularly evident under × 2 stocking density stress, where × 2 V trout retained elevated immune functions.

Consistent with earlier reports^76,77^, EE did not affect growth, hepato- and gut-somatic indices, or condition factor. The splenosomatic index (SSI) was lower in the V setup during Exp1, possibly due to reduced inflammation or erythropoietic shifts^78^. SSI positively correlated with granulocyte counts, suggesting spleen size reflects immune cell turnover. Under × 2 stocking density, SSI did not differ significantly between setups, but a slight increase (*p* > 0.05) was noted under × 2 conditions. Such increases in SSI was noted in previous studies as a compensatory response to stressful events like hypoxia or ammonia buildup^79^.

From an immunological perspective, the V setup provided a more desirable profile than the H or C setups. Although the H setup enhanced certain immune markers, it also induced signs of hyperactivation and potential stress-related damage. In contrast, the V setup supported immune stimulation without excessive inflammation. Importantly, beyond immunological outcomes, enrichment strategies must be feasible for real-world aquaculture. In this regard, the V setup is more practical: it avoids the debris accumulation seen in gravel-based H setups and is easier to clean and maintain. These practical advantages further support the suitability of vertical enrichment during this phase of the rearing cycle.

The positive effects of the V setup echo evolutionary patterns, where fish evolved in complex habitats with structural refuge^80^. This evolutionary reliance on shelter is deeply embedded in fish behaviour. Even predators exploit the shelter seeking behaviour of fish. For example, black egrets form umbrella-like structures with their wings to attract fish seeking refuge, thereby improving their hunting success^81^. EE may tap into these ingrained behavioural and physiological responses that modulate immune function. While sustained swimming activity and water flow are known to influence stress physiology and immune competence in salmonids^82,83^, the present study did not manipulate water current or exercise regimes. Importantly, significant enrichment × stocking density interactions observed for several parameters suggest that the effects of vertical enrichment are context dependent and may be mediated by altered social dynamics or space use under different density conditions rather than by exercise per se.

In practical terms, enrichment is rarely implemented in commercial aquaculture^84,85^, partly due to the lack of direct productivity benefits. This study demonstrates that EE, particularly with vertical cords, can act as a remote immunostimulant, potentially replacing or complementing feed- or injection-based immunostimulants. From an applied perspective, simple vertical structures such as suspended cords or strips represent a potentially scalable form of enrichment that could be adapted to larger production systems, including tanks ≥ 1 m^3^, with relatively low material cost and minimal interference with feeding, water flow, or routine husbandry. However, given that behavioural and physiological responses to enrichment may vary depending on tank dimensions, spatial configuration, and system design, the effectiveness of such structures should be evaluated within the context of specific rearing environments. Nevertheless, the use of low-cost, passive vertical enrichment offers a promising framework for improving fish welfare and immune competence without substantial increase in production costs, supporting its consideration in commercial aquaculture systems.

## Conclusion and future outlook

This study demonstrates that structural EE, especially vertical cords, modulates the neuroendocrine-immune axis in rainbow trout. It enhances immune functionality through behavioural and physiological pathways without requiring invasive treatments. The V setup maintained its immunostimulatory effect under × 2 stocking density, suggesting scalability and robustness. Given its practicality and immune benefits, vertical EE holds promise as a welfare-improving and health-promoting intervention in intensive aquaculture systems.

Future studies should validate these findings through pathogen challenge trials. Mechanistic exploration should target both direct neuroimmune pathways, such as GABAergic signalling^86^, and indirect routes involving host–microbiota interactions^87^, to delineate the immunostimulatory mode of action of environmental enrichment. Incorporating structural enrichment into standard aquaculture protocols may yield a dual benefit: improved fish welfare and reduced reliance on chemical immunostimulants.

## Supplementary Information

Below is the link to the electronic supplementary material.

Supplementary Material 1

## Data Availability

The Datasets, R scripts, and statistical test results used in this study will be available on the OpenAgrar server ([https://www.openagrar.de/]) upon publication, further data are available from the corresponding author (Jörn Peter Scharsack) on reasonable request.
