# Supplementary material for: Environmental enrichment as an immunostimulant for rainbow trout aquaculture
Source: Sci Rep. 2026 Apr 9;16:12367. doi: 10.1038/s41598-026-44702-2 (PMC13079716; doi:10.1038/s41598-026-44702-2)
Supplement: Supplementary file 1 — Supplementary Material 1 [file 41598_2026_44702_MOESM1_ESM.docx]

**Supplementary data**


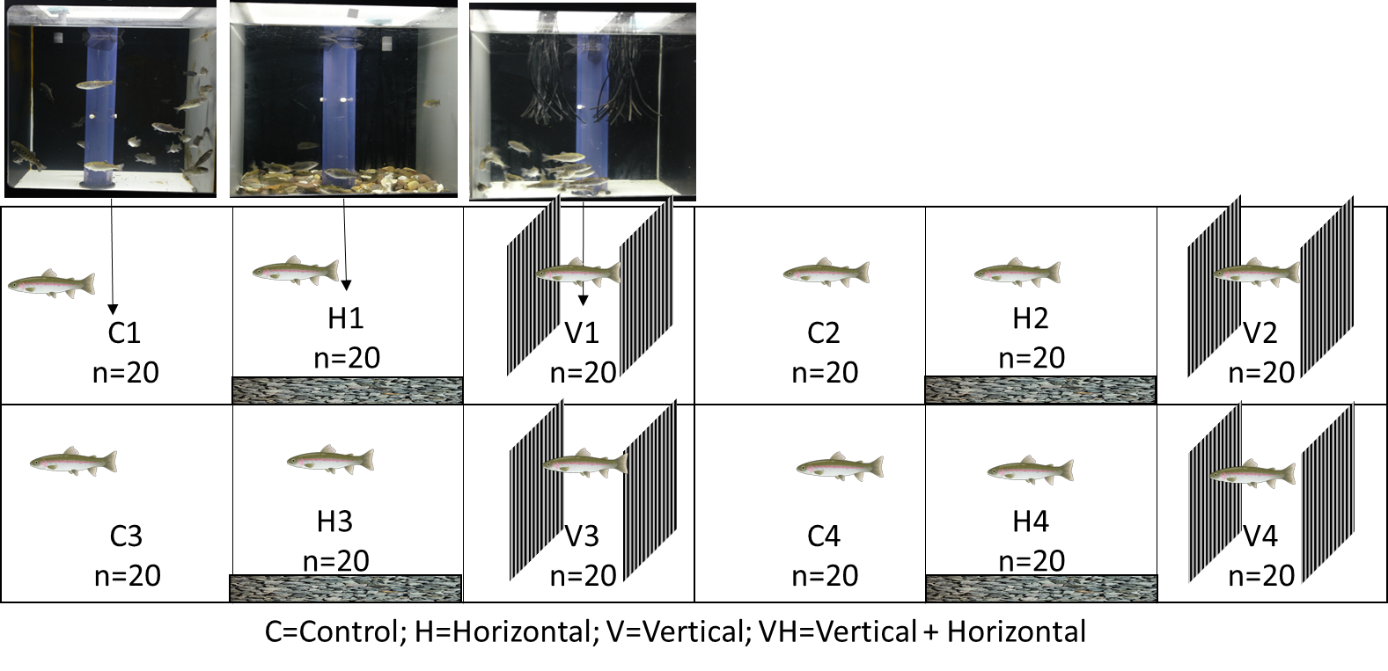


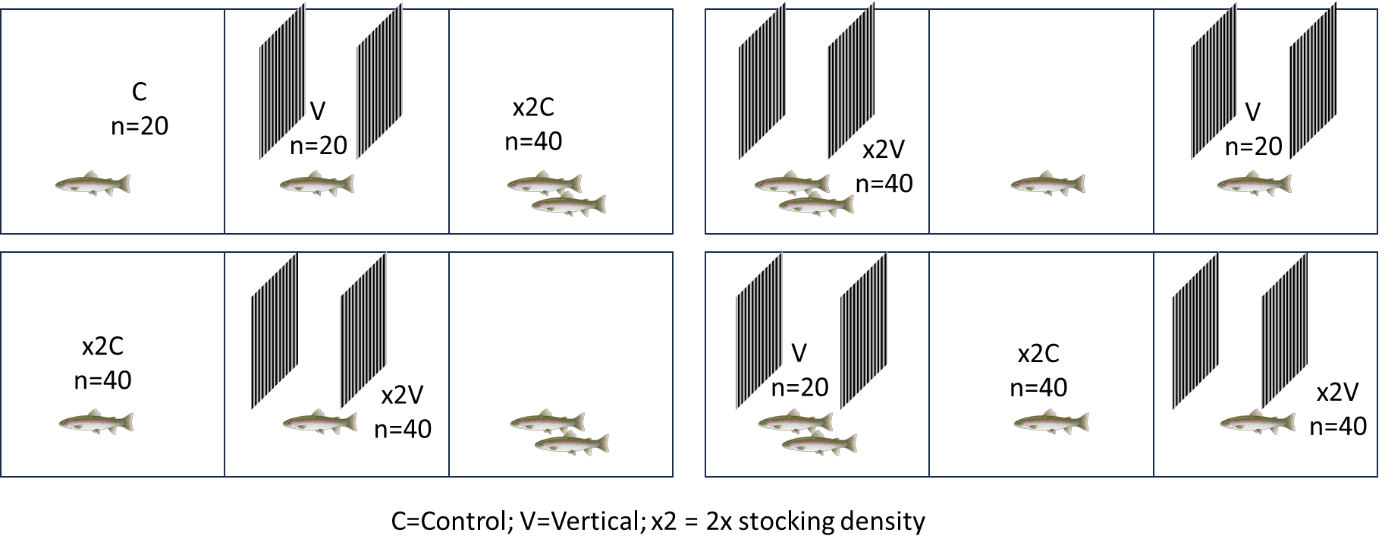


**Supplementary figure 1.** illustrates the positioning of environmental enrichment, control setup, and replicates. The inset shows an actual picture of the experimental aquaria. Abbreviations: C = Control, H = Horizontal, V = Vertical, ×2C = high stocking density Control, and ×2V = high stocking density Vertical.


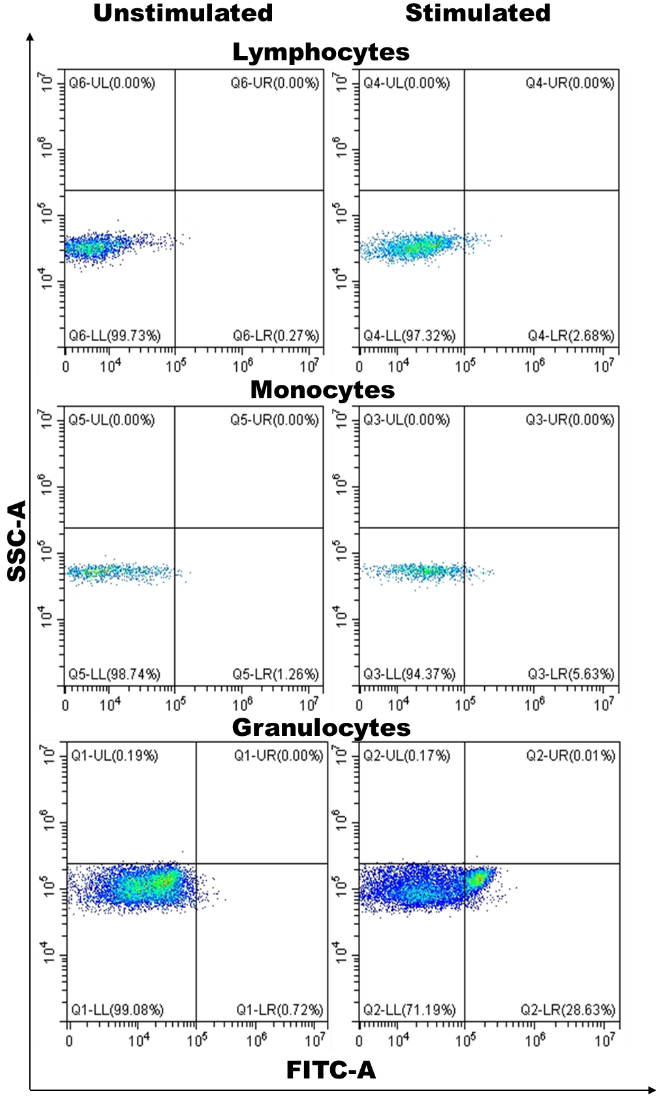


**Supplementary figure 2.** An example flow cytometry profile of DCFDA assay using freshly isolated rainbow trout head kidney leukocytes (HKL). Live cells and subpopulations of HKL were gated according to our previous protocol. Note: upon stimulation only HKL of granulocyte population is moving to quadrant 2 (higher fluorescence). This is also reflected in our correlation studies where granulocyte percentages were shown to be highly correlated to respiratory burst activity.


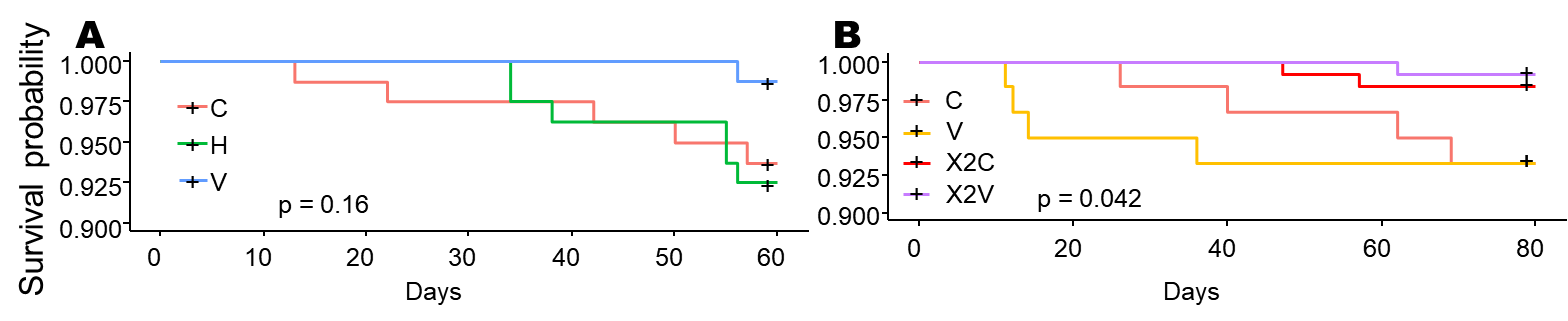


**Supplementary figure 3.** Kaplan-Meier survival curves for trout in Exp1 involving different environmental enrichment setups: C (control), H (horizontally placed gravel), and V (vertically placed plastic cords). No significant differences in survival probability (*p =* 0.16) were observed at the end of the experiment. **B.** Kaplan-Meier survival curves for trout in a high stocking density Exp2 involving C (control), V (vertically placed plastic cords), ×2C, and ×2V setups. While a significant difference in survival probability (*p =* 0.042) was observed, subsequent pairwise comparisons did not yield any significant differences (lowest *p =* 0.16).


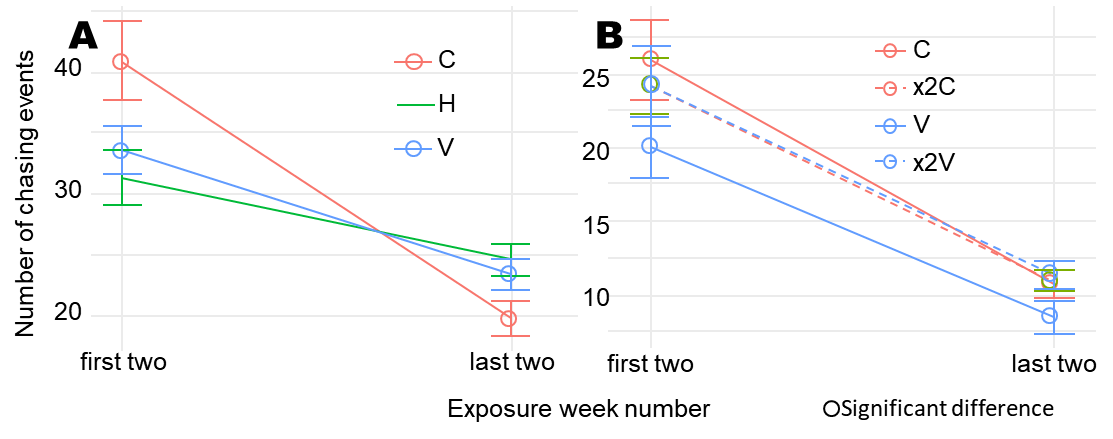


**Supplementary figure 4.** **A. Number of chasing events over time across different environmental enrichment setups: control (C), horizontal (H), and vertical (V). B. High stocking density setups: ×2C and ×2V.**

The interaction plots show how chasing events varied between the first two and last two weeks of the exposure period. In both the C and V setups, chasing events declined markedly over time, while the H setup showed a relatively stable trend with only a minimal decrease. Chasing events were recorded on all weekdays in all aquaria. A total of 38 observations per setup were recorded during the first two weeks and 48 observations per setup during the last two weeks. For the high stocking density Exp2, 33 observations were recorded for the first two weeks and 27 for the last two weeks per each setup. Data are presented as mean ± SEM. The H setup did not differ significantly between time points (p > 0.05). Note: The y-axis scale differs between panels.


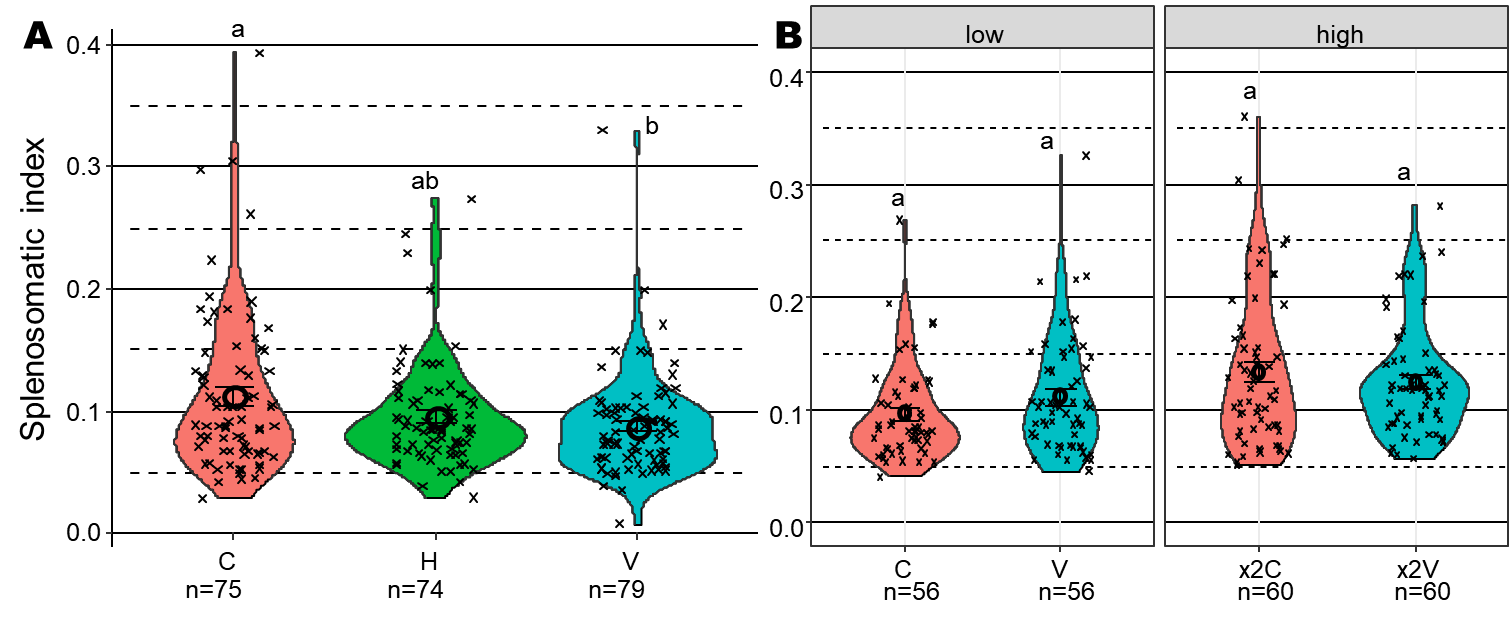


**Supplementary figure 5. Splenosomatic index (SSI) of trout exposed to different environmental enrichment setups.**

(A) SSI of trout exposed to control (C), horizontal (H), and vertical (V) setups for 60 days. (B) SSI of trout exposed to control (C), vertical (V), double stocking control (×2C), and double stocking vertical (×2V) setups for 60 days. After exposure, spleens were dissected, weighed, and SSI calculated as the ratio of spleen weight (g) to fish weight (g). Violin plots show the kernel density distribution of SSI data, with each point representing the SSI of an individual fish. Mean ± SEM is indicated by black circles and error bars. Different letters indicate significant differences between setups. Note: The y-axis scale differs between panels.

**
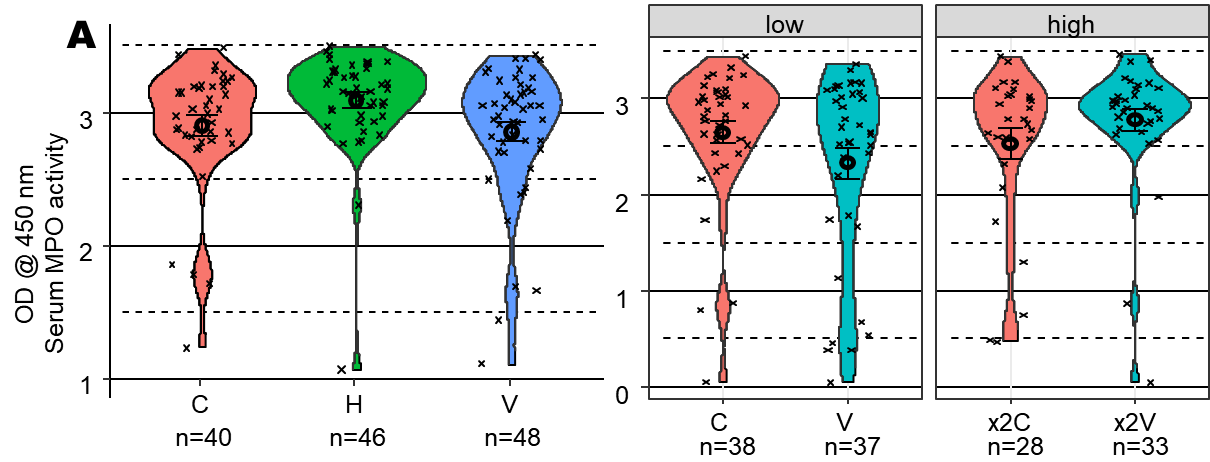
**


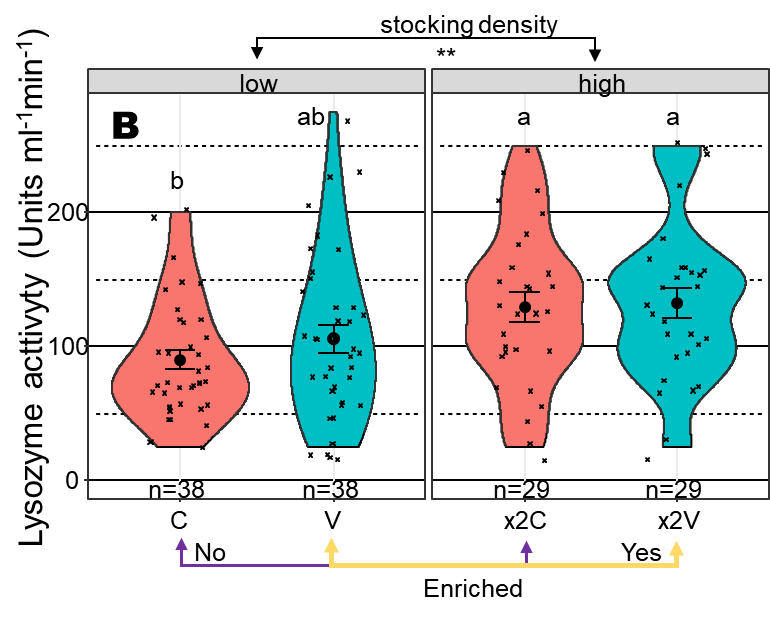


**Supplementary figure 6.** **Effect of 60 days of exposure to environmental enrichment setups on serum biomarkers in trout.**

(**A**) Serum myeloperoxidase (MPO) activity in trout exposed to control (C), horizontal (H), and vertical (V) setups. MPO was measured using the TMB (3,3',5,5'-Tetramethylbenzidine) oxidation method and reported as optical density at 450 nm (OD450). No significant differences were found, though a trend (*p <* 0.1) was observed between H and V. (**B**) Serum lysozyme activity in trout from Exp2, measured by the change in turbidity at 490 nm. Lysozyme activity was significantly influenced by stocking density, indicated by ** (*p <* 0.01). Different letters indicate significant differences (*p <* 0.05). Violin plots show the kernel density distribution of raw data, with each point representing an individual fish datum. Mean ± SEM is shown as black circles and error bars.

**
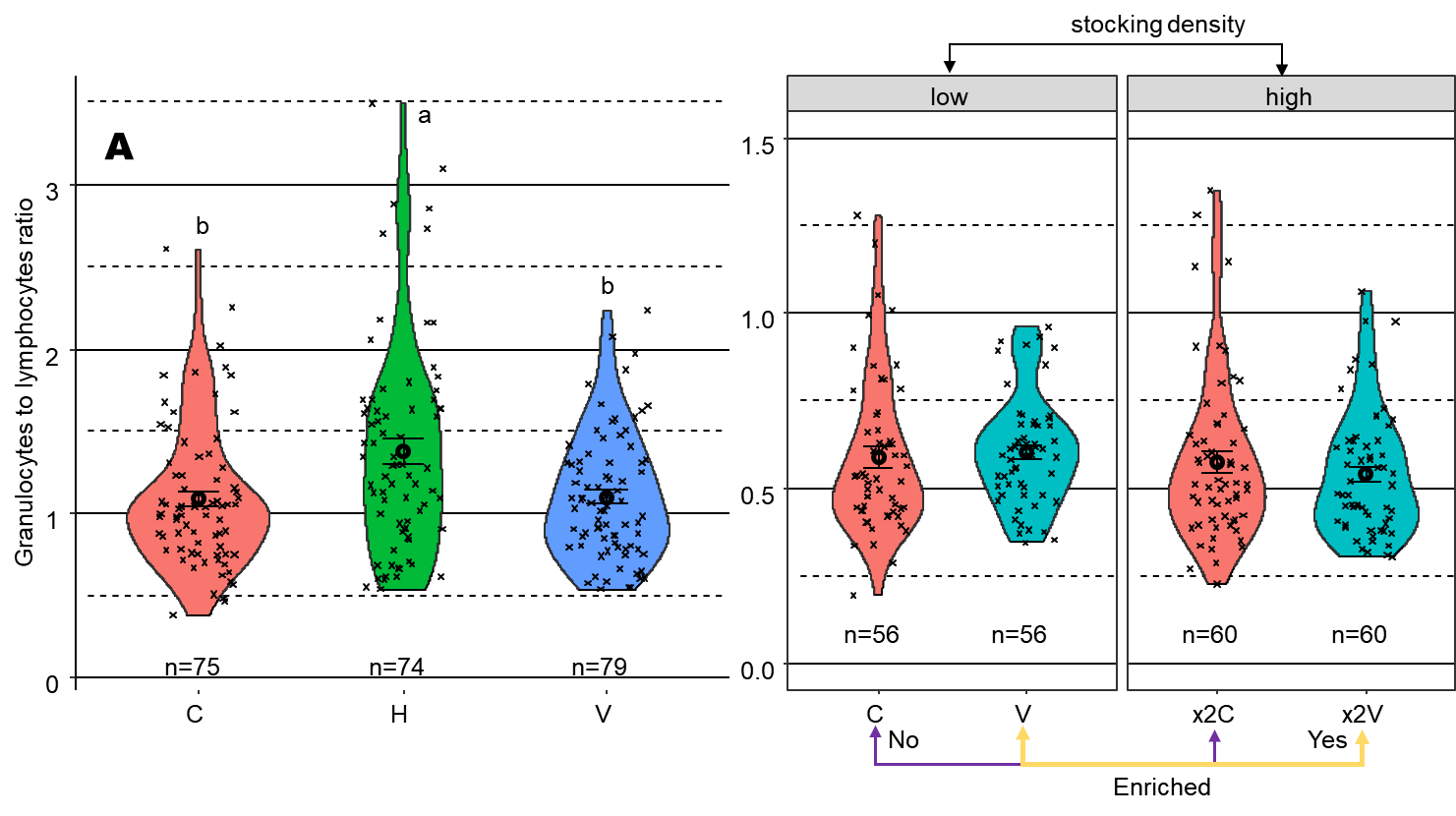
**

**
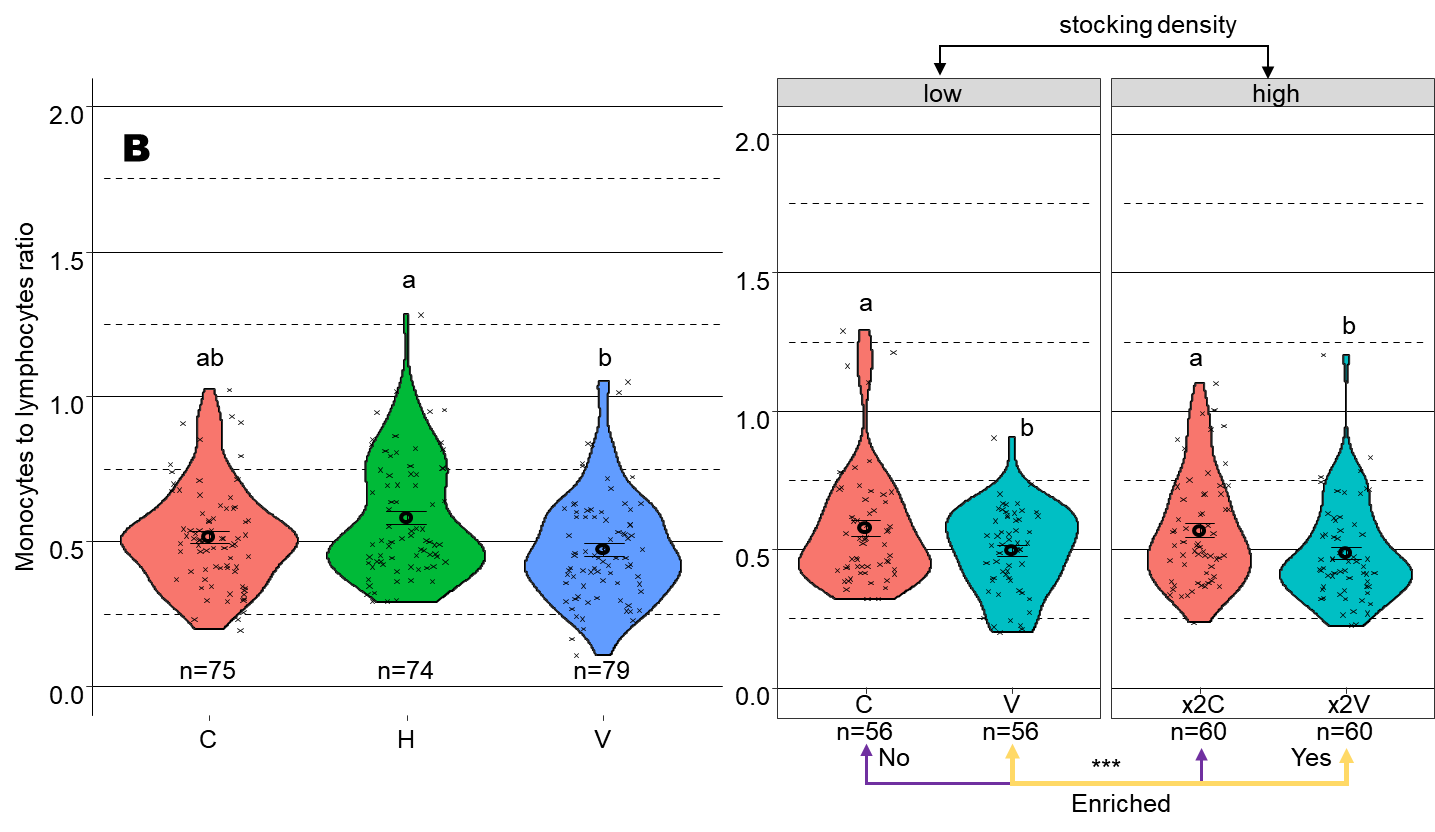
**

**Supplementary figure 7. Ratios of body weight-normalized head kidney leukocyte (HKL) count in trout exposed to environmental enrichment setups.**

Granulocyte/Lymphocyte ratio **(A)** and Monocyte/Lymphocyte ratio **(B)** in trout exposed to control (C), horizontal (H), and vertical (V) environmental enrichment setups for 60 days. After exposure, head kidneys were dissected, and leukocytes were counted using flow cytometry. Differential counts were obtained via gating, expressing the percentage of live (propidium iodide negative) HKL. Sample sizes (n) are shown under the x-axis. Violin plots show kernel density distributions of raw data, with each point representing an individual fish. Mean ± SEM is shown as black circles and error bars, respectively. Significant differences between factor levels are indicated by asterisks (*** for *p <* 0.001), and different letters denote significant setup level differences. Note: The y-axis scale differs between panels.


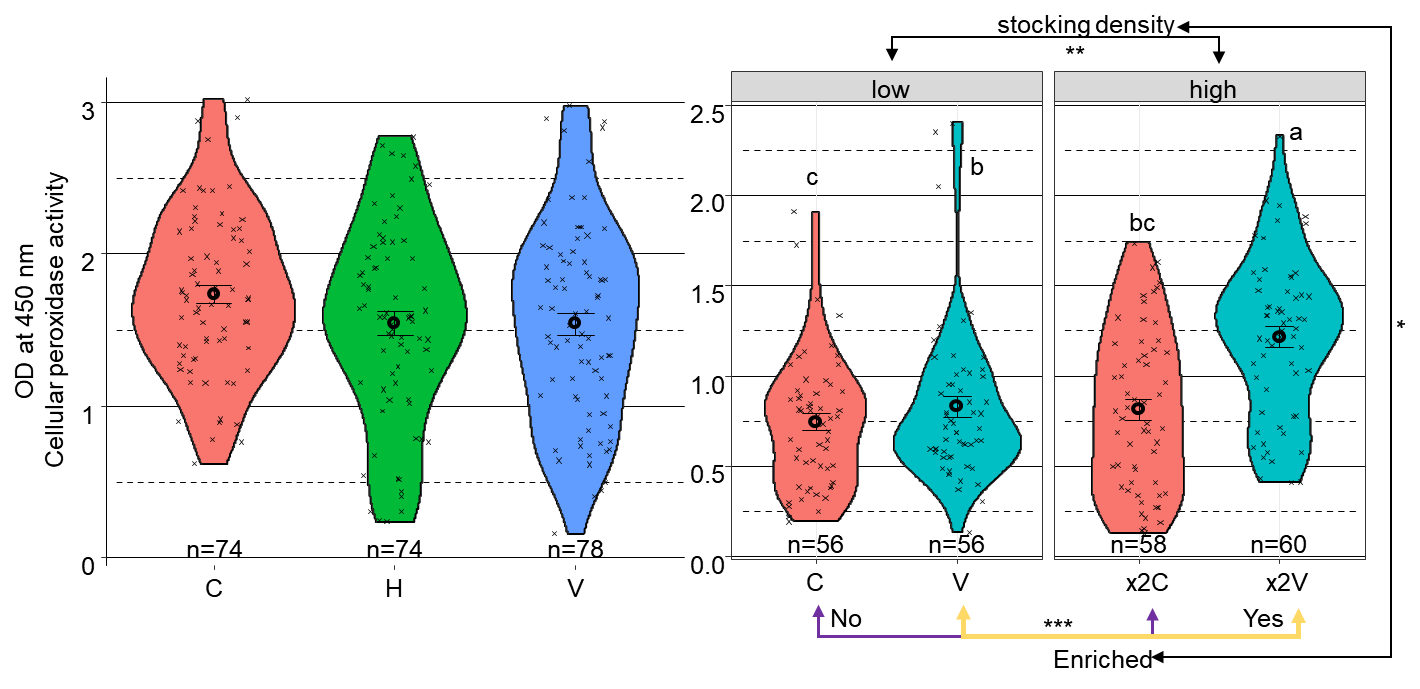


**Supplementary figure 8.** **Cellular peroxidase activity in head kidney leukocytes (HKL) of trout from different environmental setups and stocking densities.**

Cellular peroxidase activity, predominantly driven by myeloperoxidase in phagocytes, was measured colorimetrically following lysis of freshly isolated HKL. In Exp1, there were no significant differences between control (C), horizontal (H), and vertical (V) enrichment setups. However, interaction modelling between setup and granulocyte percentages revealed increased peroxidase activity in V setup trout at the mean granulocyte level (36%), and at high hypothetical granulocyte percentages, activity was significantly higher in V compared to H setup. In Exp2, interaction between enrichment and stocking density significantly influenced peroxidase levels. Both enrichment and stocking density independently affected peroxidase activity, with the enrichment effect being more pronounced at high stocking density. Violin plots show kernel density of raw values with individual data points. Mean ± SEM is indicated by black circles and bars. Asterisks denote statistical significance (** for *p <* 0.01 and *** for *p <* 0.001) between the factors (enrichment and stocking density), and different letters indicate significant differences between setup (*p <* 0.05). Note: The y-axis scale differs between panels.


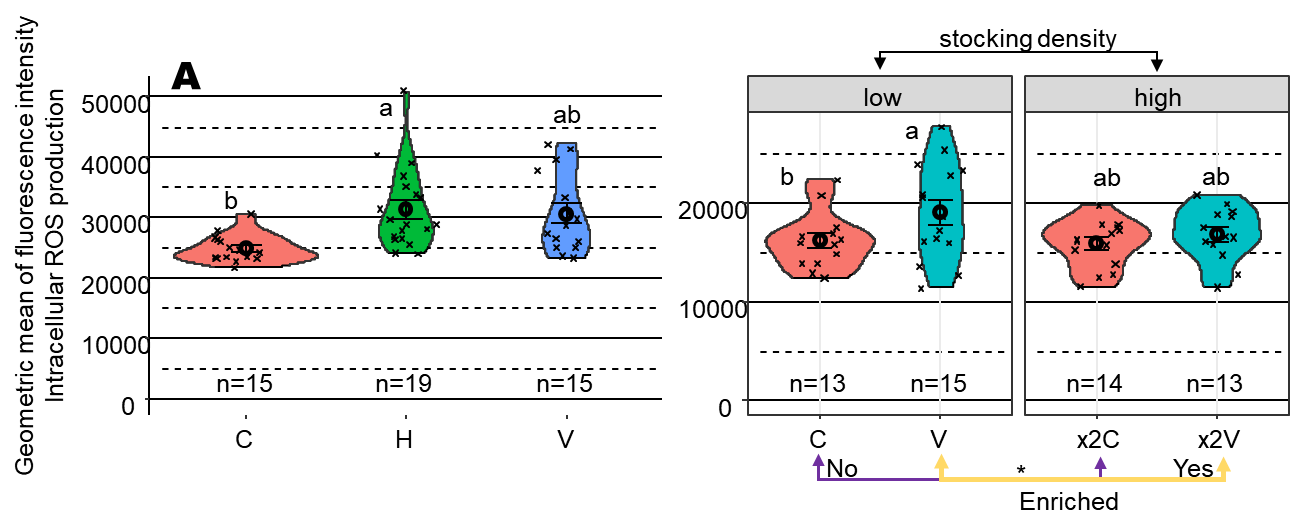


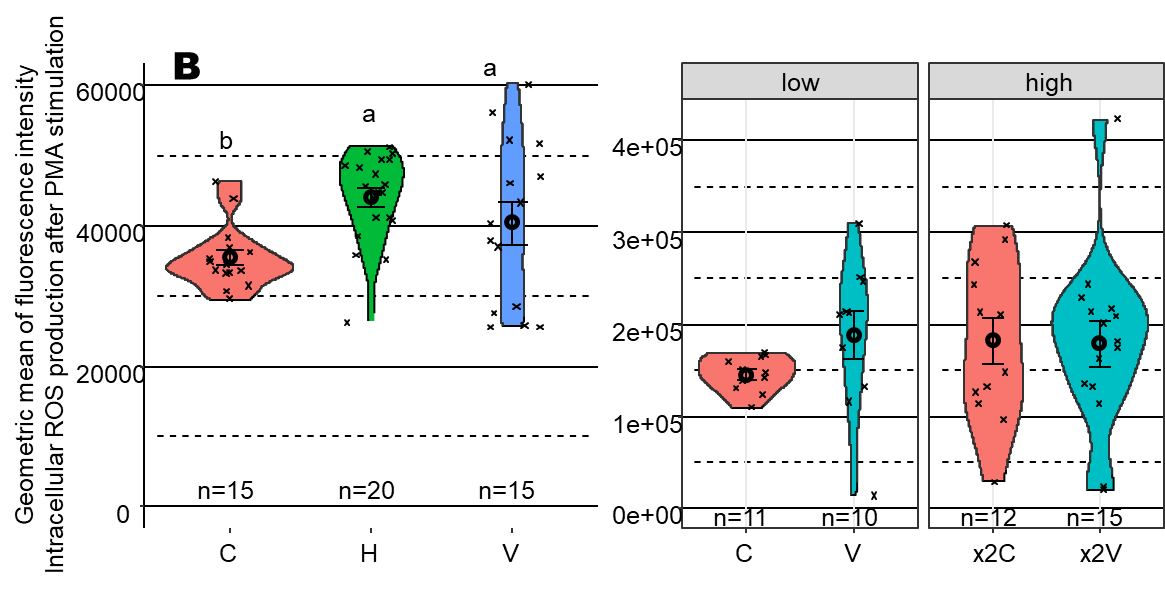


**Supplementary figure 9.** **Environmental enrichment (EE) induced enhanced production of reactive oxygen species (ROS) by head kidney leukocytes (HKL).** **A.** Unstimulated; **B.** PMA-stimulated geometric mean of DCFDA fluorescence of unstimulated HKL or PMA-stimulated HKL. Violin plots represent the kernel density distribution of the geometric mean DCFDA fluorescence intensity, while individual points (x) represent the geometric mean for each fish. Significant differences between setups are denoted by different letters (*p <* 0.05). Sample sizes are indicated as n. The mean ± SEM is shown as black circles and error bars, respectively. Note: The y-axis scale differs between panels.


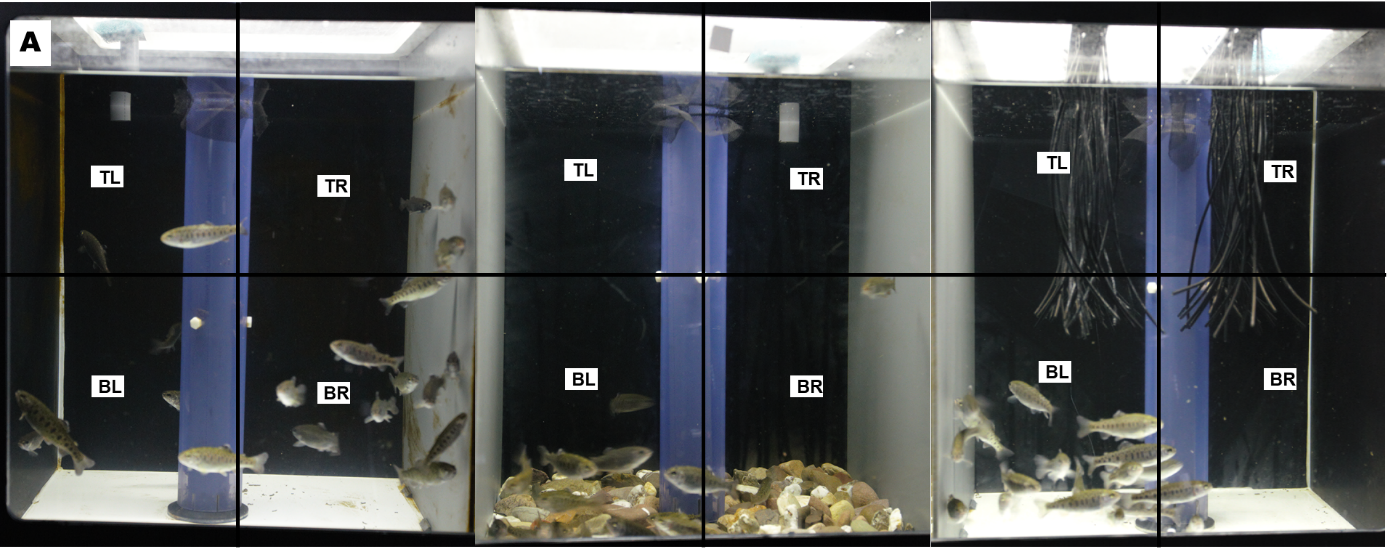


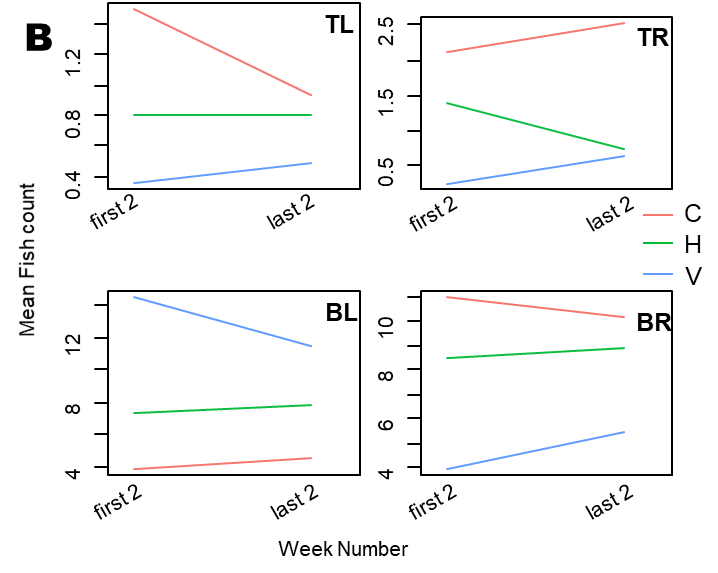


**Supplementary figure 10. A.** Quadrant analysis of experimental aquaria depicting the spatial arrangement of trout within the aquaria. Photographs of the setup were taken at short intervals on all weekdays during the first and last two weeks of the experiment. Quadrants in the photos were drawn using ImageJ, and trout within each quadrant (BL = bottom left; BR = bottom right; TL = top left; TR = top right) were then manually counted. **B.** Interaction plot of quadrant analysis data showing the mean number of trout in each quadrant. EE setup: C = control; H = horizontally placed gravel; and V = vertically placed plastic cords. Though trout appear to be highly distributed in C and confined to particular quadrants in the figure, statistical analyses did not show any significant difference.

**Supplementary video 1** showing a sample chasing event. For clarity, the individual trout chasing each other were marked with pointers, red and blue.
